# A single-nucleus transcriptomic atlas of primate testicular aging reveals exhaustion of the spermatogonial stem cell reservoir and loss of Sertoli cell homeostasis

**DOI:** 10.1093/procel/pwac057

**Published:** 2022-11-18

**Authors:** Daoyuan Huang, Yuesheng Zuo, Chen Zhang, Guoqiang Sun, Ying Jing, Jinghui Lei, Shuai Ma, Shuhui Sun, Huifen Lu, Yusheng Cai, Weiqi Zhang, Fei Gao, Andy Peng Xiang, Juan Carlos Izpisua Belmonte, Guang-Hui Liu, Jing Qu, Si Wang

**Affiliations:** Advanced Innovation Center for Human Brain Protection, National Clinical Research Center for Geriatric Disorders, Xuanwu Hospital Capital Medical University, Beijing 100053, China; Aging Translational Medicine Center, International Center for Aging and Cancer, Beijing Municipal Geriatric Medical Research Center, Xuanwu Hospital, Capital Medical University, Beijing 100053, China; University of Chinese Academy of Sciences, Beijing 100049, China; CAS Key Laboratory of Genomic and Precision Medicine, Beijing Institute of Genomics, Chinese Academy of Sciences, Beijing 100101, China; China National Center for Bioinformation, Beijing 100101, China; The Fifth People’s Hospital of Chongqing, Chongqing 400062, China; State Key Laboratory of Stem Cell and Reproductive Biology, Institute of Zoology, Chinese Academy of Sciences, Beijing 100101, China; University of Chinese Academy of Sciences, Beijing 100049, China; State Key Laboratory of Stem Cell and Reproductive Biology, Institute of Zoology, Chinese Academy of Sciences, Beijing 100101, China; University of Chinese Academy of Sciences, Beijing 100049, China; Advanced Innovation Center for Human Brain Protection, National Clinical Research Center for Geriatric Disorders, Xuanwu Hospital Capital Medical University, Beijing 100053, China; Aging Translational Medicine Center, International Center for Aging and Cancer, Beijing Municipal Geriatric Medical Research Center, Xuanwu Hospital, Capital Medical University, Beijing 100053, China; State Key Laboratory of Membrane Biology, Institute of Zoology, Chinese Academy of Sciences, Beijing 100101, China; Beijing Institute for Stem Cell and Regenerative Medicine, Beijing 100101, China; Institute for Stem cell and Regeneration, CAS, Beijing 100101, China; Aging Biomarker Consortium, China; State Key Laboratory of Membrane Biology, Institute of Zoology, Chinese Academy of Sciences, Beijing 100101, China; Beijing Institute for Stem Cell and Regenerative Medicine, Beijing 100101, China; Institute for Stem cell and Regeneration, CAS, Beijing 100101, China; Advanced Innovation Center for Human Brain Protection, National Clinical Research Center for Geriatric Disorders, Xuanwu Hospital Capital Medical University, Beijing 100053, China; Aging Translational Medicine Center, International Center for Aging and Cancer, Beijing Municipal Geriatric Medical Research Center, Xuanwu Hospital, Capital Medical University, Beijing 100053, China; State Key Laboratory of Membrane Biology, Institute of Zoology, Chinese Academy of Sciences, Beijing 100101, China; Beijing Institute for Stem Cell and Regenerative Medicine, Beijing 100101, China; Institute for Stem cell and Regeneration, CAS, Beijing 100101, China; University of Chinese Academy of Sciences, Beijing 100049, China; CAS Key Laboratory of Genomic and Precision Medicine, Beijing Institute of Genomics, Chinese Academy of Sciences, Beijing 100101, China; Institute for Stem cell and Regeneration, CAS, Beijing 100101, China; China National Center for Bioinformation, Beijing 100101, China; Sino-Danish College, University of Chinese Academy of Sciences, Beijing 101408, China; Sino-Danish Center for Education and Research, Beijing 101408, China; Aging Biomarker Consortium, China; State Key Laboratory of Stem Cell and Reproductive Biology, Institute of Zoology, Chinese Academy of Sciences, Beijing 100101, China; University of Chinese Academy of Sciences, Beijing 100049, China; Beijing Institute for Stem Cell and Regenerative Medicine, Beijing 100101, China; Institute for Stem cell and Regeneration, CAS, Beijing 100101, China; Center for Stem Cell Biology and Tissue Engineering, Key Laboratory for Stem Cells and Tissue Engineering, Ministry of Education, Sun Yat-sen University, Guangzhou 510000, China; Department of Biochemistry, Zhongshan School of Medicine, Sun Yat-sen University, Guangzhou 510000, China; Altos Labs, Inc., San Diego, CA 92121, USA; Advanced Innovation Center for Human Brain Protection, National Clinical Research Center for Geriatric Disorders, Xuanwu Hospital Capital Medical University, Beijing 100053, China; Aging Translational Medicine Center, International Center for Aging and Cancer, Beijing Municipal Geriatric Medical Research Center, Xuanwu Hospital, Capital Medical University, Beijing 100053, China; State Key Laboratory of Membrane Biology, Institute of Zoology, Chinese Academy of Sciences, Beijing 100101, China; University of Chinese Academy of Sciences, Beijing 100049, China; Beijing Institute for Stem Cell and Regenerative Medicine, Beijing 100101, China; Institute for Stem cell and Regeneration, CAS, Beijing 100101, China; Aging Biomarker Consortium, China; State Key Laboratory of Stem Cell and Reproductive Biology, Institute of Zoology, Chinese Academy of Sciences, Beijing 100101, China; University of Chinese Academy of Sciences, Beijing 100049, China; Beijing Institute for Stem Cell and Regenerative Medicine, Beijing 100101, China; Institute for Stem cell and Regeneration, CAS, Beijing 100101, China; Aging Biomarker Consortium, China; Advanced Innovation Center for Human Brain Protection, National Clinical Research Center for Geriatric Disorders, Xuanwu Hospital Capital Medical University, Beijing 100053, China; Aging Translational Medicine Center, International Center for Aging and Cancer, Beijing Municipal Geriatric Medical Research Center, Xuanwu Hospital, Capital Medical University, Beijing 100053, China; The Fifth People’s Hospital of Chongqing, Chongqing 400062, China; Aging Biomarker Consortium, China

**Keywords:** single-nucleus RNA sequencing, primate, testis, aging, WT1

## Abstract

The testis is pivotal for male reproduction, and its progressive functional decline in aging is associated with infertility. However, the regulatory mechanism underlying primate testicular aging remains largely elusive. Here, we resolve the aging-related cellular and molecular alterations of primate testicular aging by establishing a single-nucleus transcriptomic atlas. Gene-expression patterns along the spermatogenesis trajectory revealed molecular programs associated with attrition of spermatogonial stem cell reservoir, disturbed meiosis and impaired spermiogenesis along the sequential continuum. Remarkably, Sertoli cell was identified as the cell type most susceptible to aging, given its deeply perturbed age-associated transcriptional profiles. Concomitantly, downregulation of the transcription factor Wilms’ Tumor 1 (WT1), essential for Sertoli cell homeostasis, was associated with accelerated cellular senescence, disrupted tight junctions, and a compromised cell identity signature, which altogether may help create a hostile microenvironment for spermatogenesis. Collectively, our study depicts in-depth transcriptomic traits of non-human primate (NHP) testicular aging at single-cell resolution, providing potential diagnostic biomarkers and targets for therapeutic interventions against testicular aging and age-related male reproductive diseases.

## Introduction

The testis plays a crucial role throughout life in spermatogenesis and production of male sex hormones, pivotal processes in male fertility and multiple physiological functions in men (Oatley and Brinster, 2012; Finkelstein et al., 2013). In aging, spermatogenesis undergoes progressive decline, a leading cause of male infertility that also increases the risk for genetic or other types of abnormalities in offspring (Basaria, 2013). In addition, testicular aging is accompanied by changes in sex hormone metabolism, which may contribute to a series of male reproductive diseases (i.e., late-onset hypogonadism [LOH], erectile dysfunction and so on), reducing quality of life in the elders (Sampson et al., 2007). Therefore, acquiring a comprehensive mechanistic understanding of primate testicular aging remains an outstanding challenge.

The testicular parenchyma contains highly convoluted seminiferous tubules and the surrounding interstitial compartment (Kaur et al., 2014). The seminiferous epithelium consists of Sertoli cells, structural support cells, and a stratified germinal epithelium layer, in which germ cells progress through spermatogonium, spermatocyte and haploid spermatid developmental stages (Dym and Fawcett, 1970; Dym, 1973; Kaur et al., 2014). Sertoli cell is an essential component of the germ cell niche that instructs germ cell progression to spermatozoa by cell–cell contact and by controlling the extracellular microenvironment (Griswold, 1998; O’Donnell et al., 2022). Within the testicular interstitium, Leydig cells, which secrete testosterone to support steroidogenesis, and other vascular cells, such as vascular smooth muscle cells (VSMCs) and endothelial cells (ECs), are the major cell types (Heinrich and DeFalco, 2020). Although testicular aging is a heterogenous process, it has been documented as a set of histomorphological changes (Paniagua et al., 1991; Perheentupa and Huhtaniemi, 2009), including seminiferous tubule dilatation, depletion of germ cells, decrease in the numbers of Sertoli cells and Leydig cells, arteriolar sclerosis and increased interstitial fibrosis, all of which are associated with age-dependent decline in spermatogenesis and steroidogenesis (Johnson et al., 1987; Perheentupa and Huhtaniemi, 2009; Gunes et al., 2016; Kaufman et al., 2019; Zhao et al., 2019; Matzkin et al., 2021; Wang et al., 2022b). However, the cellular and molecular characteristics underlying primate testicular aging are much less well understood.

Given ethical restrictions, obtaining disease-free human testicular biopsies from both young and aged individuals to carry out an unbiased study of testicular aging is challenging. However, given that non-human primates (NHPs) share similar genetic, physiological, and particularly spermatogenic features with humans, e.g., the spermatogonia subtypes and the mechanism for the first wave of spermatogenesis (Fayomi and Orwig, 2018), they represent ideal models for the study of primate testicular aging. In particular, the highly complex cellular composition of the testis requires dissection of molecular mechanisms at single-cell resolution (Zhong et al., 2020; Fang et al., 2022; Zou et al., 2022), i.e., single-cell/nucleus RNA sequencing (scRNA-seq/snRNA-seq) techniques that have already revealed age-related transcriptional alterations underlying the heterogeneous process of aging and age-related diseases at cell type-specific resolution in multiple organs (Li et al., 2021; Ma et al., 2020, 2021, 2022; Wang et al., 2021a; Zhang et al., 2020, 2021, 2022; Zhong et al., 2020; Zou et al., 2021; Cai et al., 2022). However, snRNA-seq has not yet been applied to resolve the cellular and molecular signatures of testicular aging in the primates.

In the present study, we demonstrated an array of aging-associated damages in NHP testes, including increased fibrosis in interstitium and basal membrane, age-associated lipofuscin, and lipid deposits, as well as pronounced accumulation of senescent cells. We also generated a high-resolution single-nucleus transcriptomic landscape of testicular aging in NHPs. This atlas enabled us to identify transcriptional alterations underlying early-onset dysregulation in spermatogenesis, and unveil WT1-silencing as a factor disrupting Sertoli cell homeostasis and creating a consequent hostile microenvironment for spermatogenesis in the aged testis. Collectively, our study offers a valuable resource for the identification of new diagnostic biomarkers and potential therapeutic targets for the development of countermeasures against testicular aging and related reproductive diseases in men.

## Results

### Accumulation of aging-associated damages in the aged testes from cynomolgus monkeys

**Figure 1.**
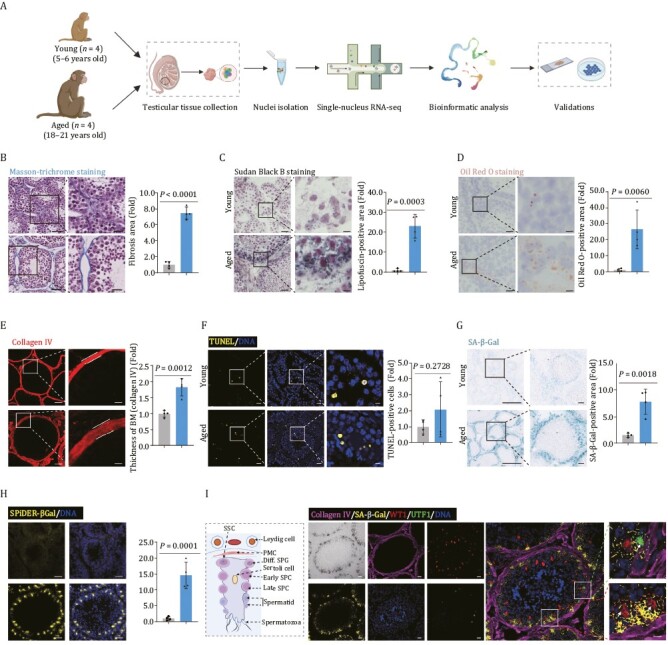
**Aging-related phenotypes of the cynomolgus monkey testes.** (A) Flow chart of experimental design of testicular aging study in cynomolgus monkeys. (B) Masson-trichrome staining analysis of testicular tissues from young and aged monkeys. Representative images are shown on the left. Quantitative data are shown as means ± SEM on the right. Fibrosis area is quantified as fold changes (Aged vs. Young). Young, *n* = 4; aged, *n* = 4 monkeys. Scale bars, 50 μm and 25 μm (zoomed-in image). (C) Sudan black B staining of testicular tissues from young and aged monkeys. Representative images are shown on the left. Quantitative data are shown as means ± SEM on the right. Lipofuscin-positive area is quantified as fold changes (Aged vs. Young). Young, *n* = 4; aged, *n* = 4 monkeys. Scale bars, 50 μm and 12.5 μm (zoomed-in image). (D) Oil Red O staining of testicular tissues from young and aged monkeys. Representative images are shown on the left. Quantitative data are shown as means ± SEM on the right. Oil Red O-positive area is quantified as fold changes (Aged vs. Young). Young, *n* = 4; aged, *n* = 4 monkeys. Scale bars, 50 μm and 12.5 μm (zoomed-in image). (E) Immunofluorescence analysis of type IV Collagen in basal membrane of seminiferous tubules. Representative images are shown on the left. Quantitative data are shown as means ± SEM on the right. Thickness of basement membrane (BM, marked by collagen IV) is quantified as fold changes (Aged vs. Young). Young, *n* = 4; aged, *n* = 4 monkeys. Scale bars, 20 μm and 5 μm (zoomed-in image). (F) TUNEL analysis of apoptotic cells in testicular tissues from young and aged monkeys. Representative images are shown on the left. TUNEL-positive cells are quantified as fold changes (Aged vs. Young) and shown on the right. Young, *n* = 4; aged, *n* = 4 monkeys. Scale bars, 20 μm and 5 μm (zoomed-in image). (G) SA-β-Gal staining of testicular tissues from young and aged monkeys. Representative images are shown on the left. SA-β-Gal-positive areas are quantified as fold changes (Aged vs. Young), and shown as means ± SEM on the right. Young, *n* = 4; aged, *n* = 4 monkeys. Scale bar, 300 μm and 50 μm (zoomed-in image). (H) SPiDER-βGal staining of testicular tissues from young and aged monkeys. Representative images are shown on the left. SPiDER-βGal-positive areas in the tissues are quantified as fold changes (Aged vs. Young), and shown as means ± SEM on the right. Young, *n* = 4; aged, *n* = 4 monkeys. Scale bar, 20 μm (zoomed-in image). (I) Co-stainings of SA-β-Gal, collagen IV, UTF1, and WT1 in testicular tissues from aged monkeys. A schematic of the germinal epithelium of seminiferous tubule is shown on the left. Representative images are shown on the right. Scale bars, 20 μm and 5 μm (zoomed-in image).

To dissect the phenotypic and molecular characteristics of NHP testicular aging, we obtained testes from four young (5–6 years old, equivalent to juvenile in human age) and four aged (18–21 years old, equivalent to approximate seventy-year-old in human age) male cynomolgus monkeys (Fig. 1A) (Ma et al., 2020, 2021; Li et al., 2021; Zhang et al., 2021, 2022). First, we assessed morphological alterations across the cohort. Although complete spermatogenesis occurred in aged testes, we observed a set of age-associated changes, which may underlie progressive testicular degeneration (Fig. 1B–H). First, we found that fibrosis in the interstitium and basal membrane of aged testes was pronounced relative to their young counterparts (Fig. 1B). Then, we observed that levels of age-dependent lipofuscin pigment and lipid deposition, classical hallmarks for testicular aging (Miquel et al., 1978; Paniagua et al., 1991), were augmented in aged testes (Fig. 1C and 1D). Importantly, the thickness of the basement membrane (BM) layer was increased, as reflected by accumulated deposition of type IV collagen-marked extracellular matrix (ECM) outside of the seminiferous tubules, in aged testes relative to young tissues (Figs. 1E and S1A). From this follows that the stiffness of the ECM increases, which impairs nutrition exchange with cells inside of seminiferous tubules (Siu and Cheng, 2008; Nie et al., 2022). Of note, we found a remarkable accumulation of senescent cells but not apoptotic cells in the aged testes, and these cells surrounded the inner edge of the basal membrane of seminiferous tubules (Fig. 1F–H). When we next combined SA-β-Gal staining with immunofluorescence staining of cell type-specific markers, we found that most SA-β-Gal-positive cells also expressed the Sertoli cell marker WT1, and others co-stained with the spermatogonial stem cell marker UTF1 (Figs. 1I, S1B and S1C), implying their susceptibility to aging. Collectively, through histological analysis, we characterized multifaceted aging-related tissue damages during NHP testicular aging that help us pinpoint onset of testicular degeneration.

### Establishment of a single-nucleus transcriptomic atlas of NHP testicular aging

**Figure 2:**
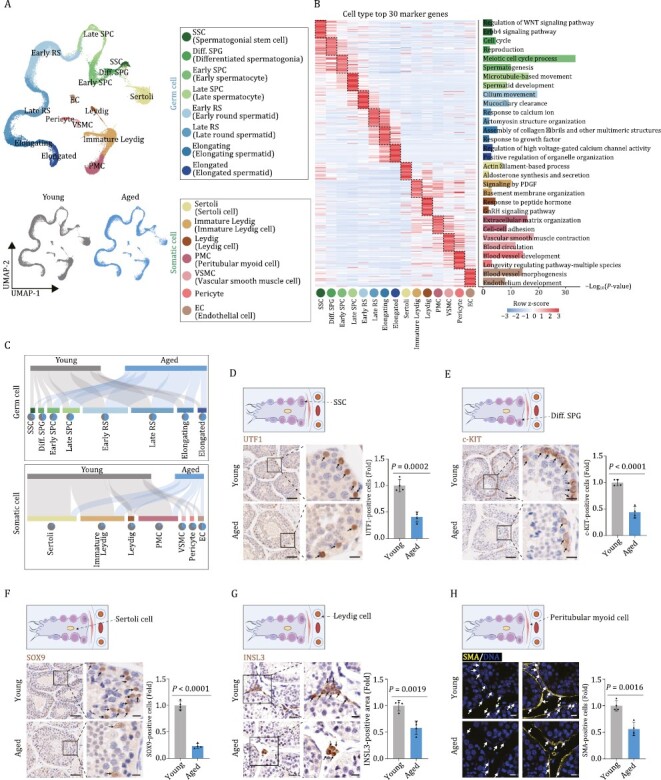
**Single-nucleus transcriptome profiling of young and aged cynomolgus monkey testes.** (A) Uniform manifold approximation and projection (UMAP) plot showing the cell types of monkey testes. Top, UMAP plot showing the 15 cell types of monkey testes. Bottom, UMAP plots showing distribution of different cell types in the young (left) and aged (right) testes. (B) Heatmap showing the expression profiles of top 30 cell type-specific marker genes of different cell types in the monkey testes with their enriched functional annotations on the right. Left, each column represents one cell type and each row indicates the expression level of one marker gene. Right, enriched GO terms for each cell type. (C) Sankey plots showing the changes of proportion across different cell types between young and aged groups. Top, germ cell group; bottom, somatic cell group. (D) Immunohistochemistry analysis of UTF1 in testicular tissues from young and aged monkeys. Representative images are shown on the left. UTF1-positive cells are quantified as fold changes of their numbers (Aged vs. Young), and shown as means ± SEM on the right. Young, *n* = 4; aged, *n* = 4 monkeys. Scale bars, 50 μm and 12.5 μm (zoomed-in image). (E) Immunohistochemistry analysis of c-KIT in testicular tissues from young and aged monkeys. Representative images are shown on the left. c-KIT-positive cells are quantified as fold changes of their numbers (Aged vs. Young), and shown as means ± SEM on the right. Young, *n* = 4; aged, *n* = 4 monkeys. Scale bars, 50 μm and 12.5 μm (zoomed-in image). (F) Immunohistochemistry analysis of SOX9 in testicular tissues from young and aged monkeys. Representative images are shown on the left. SOX9-positive cells are quantified as fold changes of their numbers (Aged vs. Young),and shown as means ± SEM on the right. Young, *n* = 4; aged, *n* = 4 monkeys. Scale bars, 50 μm and 12.5 μm (zoomed-in image). (G) Immunohistochemistry analysis of INSL3 in testicular tissues from young and aged monkeys. Representative images are shown on the left. INSL3-positive areas are quantified as fold changes (Aged vs. Young), and shown as means ± SEM on the right. Young, *n* = 4; aged, *n* = 4 monkeys. Scale bars, 25 μm and 12.5 μm (zoomed-in image). (H) Immunofluorescence analysis of SMA expression in testicular tissues from young and aged monkeys. Representative images are shown on the left. SMA-positive cells are quantified as fold changes of their numbers (Aged vs. Young), and shown as means ± SEM on the right. Young, *n* = 4; aged, *n* = 4 monkeys. Scale bars, 20 μm.

To analyze cell type-specific gene expression alterations during testicular aging, we performed snRNA-seq on testicular issues from four young and four aged cynomolgus monkeys (Fig. 1A). After stringent cell filtration, we retained a total of 70,400 high-quality single-nucleus transcriptomes for downstream analyses (Fig. 2A). To characterize testicular cell types, we mapped the gene expression profiles of well-known cell type-specific markers in the uniform manifold approximation and projection (UMAP) plot (Figs. 2A, S2A and S2B) and identified 15 major cell types, including eight types of germ cells. Germ cells included spermatogonial stem cell (*GFRA1*^+^, *UTF1*^+^, SSC), differentiated spermatogonium (*DMRT1*^+^, diff. SPG), early and late spermatocyte (*RAD51AP2*^+^, *DMC1*^+^, *SPDYA*^+^, Early/Late SPC), early and late round spermatid (*SPAG6*^+^, *CATSPER3*^+^, *RUNX2*^+^, Early/Late RS), and elongating and elongated spermatid (*TNP2*^+^, *PRM2*^+^, Elongating/Elongated). We also identified seven somatic cell types, such as Sertoli cell (*WT1*^+^, Sertoli), immature Leydig cell (*DCN*^+^, *COL1A2*^+^, Immature Leydig), Leydig cell *(INSL3*^+^, Leydig), peritubular myoid cell (*MYH11*^+^, PMC), pericyte (*PGS5*^+^, *MYO1B*^+^, Pericyte), vascular smooth muscle cell (*TAGLN*^+^, VSMC), and endothelial cell (*VWF*^+^, *PECAM1*^+^, EC) (Fig. S2C). By using functional enrichment analysis of the top 30 marker genes of each cell type following previous works (Ma et al., 2021, 2022; Wang et al., 2021a; Zhang et al., 2021), we matched the transcriptional features to their unique physiological functions (Fig. 2B). For example, the early spermatocyte marker genes aligned with the meiotic cell cycle, whereas marker genes for elongated spermatid aligned with regulation of high voltage-gated calcium channel activity (Fig. 2B).

The single-nucleus transcriptomic atlas allows us to unveil cell type-specific changes during testicular aging (Fig. 2A). When we assessed whether testicular cell populations declined with age, we first observed a decreased trend in the number of spermatogonia (Figs. 2C and S2D), an observation we experimentally validated by declined numbers of UTF1-positive SSCs and proliferative c-KIT-positive diff. SPGs in aged testes (Fig. 2D and 2E). These data are consistent with accelerated exhaustion of the stem/progenitor cell reservoir accompanied by impaired differentiation potential of SSC with age. We also detected a massive loss of Sertoli cells (marked with SOX9), Leydig cells (marked with INSL3), and PMCs (marked with SMA) (Fig. 2F–H), implying a dysregulated testicular microenvironment. Overall, an extensive aging-associated loss of critical testicular cells may fundamentally contribute to testicular degeneration.

### Transcriptomic signatures pinpoint aging-related cellular and molecular alterations in the NHP testes

**Figure 3.**
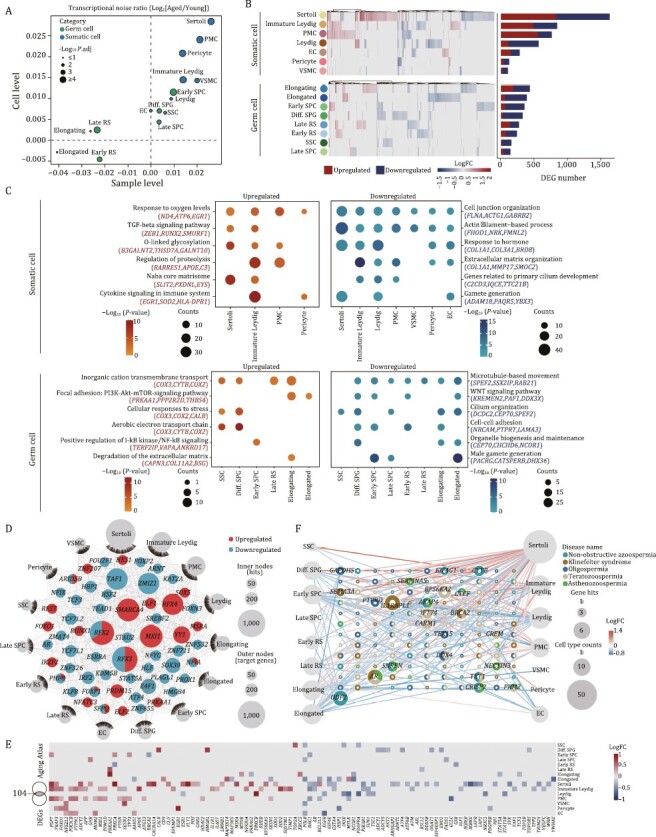
**Cell type-specific and aging-related transcriptomic signatures in NHP testes.** (A) Scatter plot showing the log_2_ ratio of transcriptional noise in the aged group compared to the young group calculated using sample averages (*n* = 8) or single cells on the *X* and *Y* axes, respectively. (B) Heatmaps and bar plots showing the expression levels and numbers of aging-related DEGs. Left, heatmaps showing the expression levels of aging-related DEGs across somatic and germ cell types. Each row represents one cell type and each column represents one aging-related DEG. Right, bar plots showing the numbers of upregulated and downregulated aging-related DEGs across cell types. (C) Dot plots showing the representative GO terms enriched for aging-related DEGs across somatic or germ cell types in the monkey testes. (D) Network visualization of upregulated and downregulated core regulatory TFs in 15 different testis cell types in the aged group compared to the young group. Internodes represent core TFs and node size positively correlates with the number of target genes regulated by specific TF. Outer nodes represent cell types and node size positively correlates with the number of target genes differentially expressed in corresponding cell types, respectively. (E) Heatmap showing the expression levels of aging-related DEGs associated with Aging Atlas database across different cell types in monkey testes. (F) Network plot showing DEGs associated with testicular diseases in different cell types of the monkey testes. The node size of cell types positively correlates with the number of DEGs in indicated cell types. The node size of genes positively correlates with the number of cell types differentially expressed in and the number of diseases contributes to. The outer ring of each gene node represents different testicular disease types, respectively.

Since increased transcriptional heterogeneity and perturbance is considered as one of the hallmark features of mammalian aging (Ma et al., 2020, 2021; Li et al., 2021; Zhang et al., 2021), we next assessed transcriptional noise during NHP testicular aging. When we calculated age-associated coefficient of variation (CV), we found that the highest cell-to-cell transcriptional perturbance relative to other populations was in aged Sertoli cells (Fig. 3A). Ranked as the next top five cell types were PMCs, pericytes, immature Leydig cells and VSMCs (Fig. 3A). We next analyzed aging-related differentially expressed genes (DEGs) (averaged Log|FoldChange| > 0.25 and adjusted *P* values < 0.05) in the aged group compared to the young group across different cell types, and noticed that aged Sertoli cell, immature Leydig cell and PMC featured the largest numbers of DEGs (820, 484 and 551 upregulated genes, 807, 356 and 208 downregulated genes, respectively) (Figs. 3B, S3A and S3B). These results underscore that somatic cells, and in particular Sertoli cells, are highly susceptible to aging, likely causing dysregulation of the germ cell development and spermatogenesis.

Next, through functional annotation enrichment analysis, we aimed to dissect the molecular pathways most affected by aging across testicular cell types. For somatic cells, we found that cell junction organization and response to hormone converged for downregulated DEGs (Fig. 3C), indicative of blunted supportive abilities and impaired sex hormone signaling with advanced age. By contrast, the TGF-β signaling pathway was enriched for upregulated DEGs in aged testicular somatic cells (Fig. 3C), in alignment with the previously observed increased fibrosis in aged testes (Fig. 1B). Although lower numbers of aging-related DEGs in germ cells reflected a relatively stable transcriptional state, we still noticed features underlying age-related testicular degeneration. For example, downregulated DEGs included genes broadly correlated to male gamete generation, microtubule-based movement, and cilium organization (Fig. 3C), likely causing compromised spermiogenesis with advanced age.

To explore how aging-related DEGs are modulated, we performed a transcriptional regulatory network analysis across all cell types and identified a handful of hub transcriptional regulators (Fig. 3D). Among downregulated TFs, we identified *TAF1,* encoding a member of testis-specific TBP-associated factors (tTAFs), which predominantly localizes at spermatocyte nucleoli and directs gene transcription necessary for spermatocyte entry into meiosis (Metcalf and Wassarman, 2007), *SOX30*, which is crucial for the transition from a late meiotic to a post-meiotic gene expression program and subsequent round spermatid development (Bai et al., 2018), and *HSF2,* which encodes heat shock transcription factor 2, that when mutated leads to defects in testis development and variable degrees of spermatogenic failure (Lahoud et al., 2001; Kallio et al., 2002; Wang et al., 2003). For somatic cells, we noticed a downregulation of *E4F1* (encoding E4F transcription factor 1), which is vital for Sertoli cell proliferation and fertility (Yan et al., 2020). Together, dysregulation of these core transcription factors is expected to dampen spermatogenesis and accelerate testicular aging by modulating a series of target genes.

To better understand the correlation of testicular aging-related DEGs and aging, we performed a conjoint comparative analysis of aging-related DEGs and genes from the Aging Atlas gene set (Aging Atlas, 2021) (Fig. 3E). Globally, this analysis again highlighted Sertoli cell as one of the cell types highly susceptible to aging, manifested with the most aging-risk genes (Fig. 3E). For instance, *POLB*, one of the top downregulated hotspot genes, encodes a key DNA polymerase involved in the base excision repair pathway (Zhang et al., 2016b; Kaufman and Van Houten, 2017), whose downregulation likely contributes to erosion of DNA repair and genomic instability with advanced parental age. Other downregulated genes included *NRG1*, which encodes Neuregulin 1, a factor essential for proliferation and differentiation of testicular cells including spermatogonia, Leydig cell and Sertoli cell (Oral et al., 2008; Umehara et al., 2016; Gregoire et al., 2018), and whose broad downregulation likely decreases the robustness of spermatogenesis during testicular aging. To further explore the correlation between aging and testicular disorders, we inquired how the expression levels of high-risk genes associated with testicular diseases [Non-obstructive azoospermia (complete failure of spermatogenesis), Oligospermia (low sperm count), Asthenozoospermia (low motility sperm), Teratozoospermia (abnormally shaped sperm), and Klinefelter syndrome (XXY syndrome; smaller testis/lower testosterone levels)] change during aging (Fig. 3F; Table S4). Among them, the androgen receptor-coding gene *AR*, which regulates testosterone responsiveness in Sertoli cells, was found to be linked to diseases with low-quality sperm (Wang et al., 2009). In addition, downregulation of *TET1*, encoding Ten-eleven translocation 1, essential for spermatogonia stem cell and germ cell differentiation (Huang et al., 2020), may also cause germ cell depletion. Notably, five genes (*ODF2*, *AKAP4*, *IFT74*, *CARMA*, *CRISP2*) have been reported to play important roles in sperm development and flagellar integrity (Bao et al., 2018; Fang et al., 2019; Ito et al., 2019; Lim et al., 2019; Shi et al., 2019), and their dysregulation likely augments sperm abnormality risk. Collectively, these findings deciphered cellular and molecular programs underlying NHP testicular aging with implications for human male reproductive diseases.

### Aging-related molecular profiling along the differentiation trajectories of spermatogenesis in NHP testes

**Figure 4.**
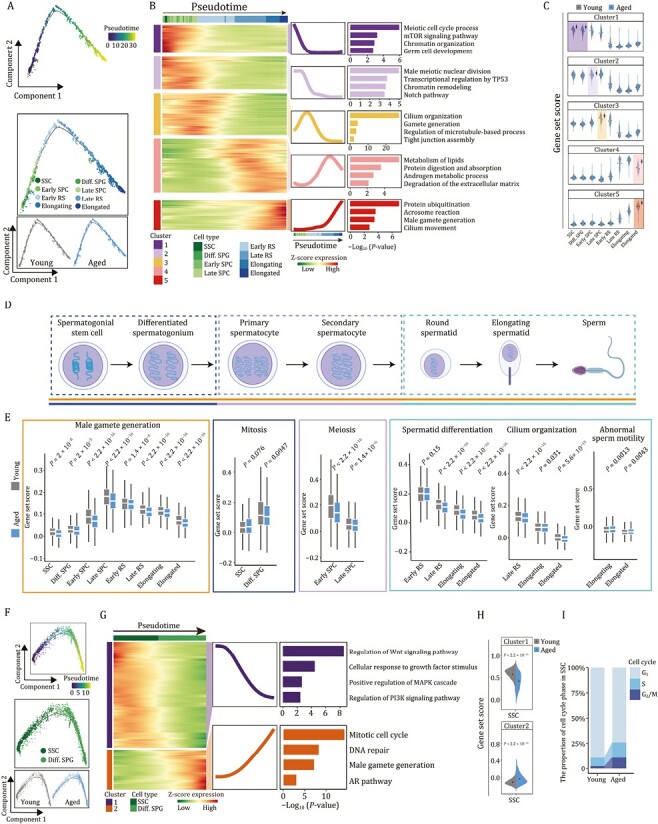
**Aging-associated molecular profiling along the differentiation trajectories of spermatogenesis in NHP testes.** (A) Pseudotime analysis of germ cells along the spermatogenesis process in monkey testes. Top, pseudotime scores of germ cells. Middle, the distribution of different germ cell types along the pseudotime trajectory. Bottom, the distribution of young and aged groups along the pseudotime trajectory. (B) Heatmaps, curve charts and bar plots showing the expression profiles of the top 1,000 genes that change along the pseudotime trajectory. According to the expression pattern, the top differentially expressed genes (DEGs) were divided into five clusters. Left, heatmap showing the expression levels of the top DEGs. Right, curve charts and bar plots showing the expression patterns and enriched GO terms of the corresponding pattern. (C) Violin plots showing the gene set scores of genes in different clusters in young and aged groups along the spermatogenesis process. (D) Schematic diagram showing the main stages of spermatogenesis. (E) Box plots showing the representative gene set scores of each spermatogenic-stage as shown in (D). (F) Pseudotime analysis of spermatogonia in monkey testes. Top, pseudotime scores of spermatogonia in monkey testes. Middle, the distribution of two spermatogonial cell types (SSC and Diff. SPG) along the pseudotime trajectory. Bottom, the distribution of young and aged groups along the pseudotime trajectory. (G) Heatmaps, curve charts and bar plots showing the expression profiles of the top 400 genes that change along the pseudotime trajectory. According to the expression pattern, the top differentially expressed genes (DEGs) were divided into two clusters. Left, heatmaps showing the expression levels of the top DEGs. Right, curve charts and bar plots showing the expression patterns and enriched GO terms of the corresponding pattern. (H) Violin plots showing the gene set scores of genes in cluster 1 and 2 in young and aged SSCs. (I) Bar plot showing the distribution of cell cycle phase in SSCs from young and aged groups.

To explore molecular fluctuations in spermatogenesis associated with age, we used pseudotime analysis to infer germ cell differentiation trajectory as a wave-like progression from SSCs, through a step-by-step sequence, to diff. SPGs, spermatocyte, round spermatid, and finally elongated spermatid (Fig. 4A). Globally view, we did not observe any obvious difference in cell-type distribution along the trajectories between the young and aged groups (Fig. 4A). Concurrently, we established the underlying molecular cascades with respect to pseudotime and defined gene expression signatures associated with the cell fate transition along the trajectory continuum to obtain five distinct gene clusters; i.e., module 1–5 (Fig. 4B). Among them, module 1 defined genes progressively downregulated with the trajectories, especially for early spermatogenic-stage cells, which were positively correlated with pathways regulating meiotic cell cycle and germ cell development (Fig. 4B). In module 2 were genes with a plateau-like sustained high expression from SSC to late SPC but declining rapidly at later stage, and that were closely correlated with chromatin remodeling and male meiotic nuclear division, suggesting vivid chromatin dynamics during mitosis and meiosis (Fig. 4B). Genes in module 3 were gradually upregulated at early stages, declined at later timepoints but peaked at the early round spermatid stage, with an enrichment of functions related to cilium organization and regulation of microtubule-based process (Fig. 4B). Genes in module 4 displayed a pattern similar to that of module 3 but peaked at the late round spermatid stage, and were characterized by lipids and androgen metabolic processes (Fig. 4B). Intriguingly, module 5 gathered genes progressively upregulated along the trajectories, especially converging on cells at the late spermatogenic-stage, with signature genes involved in cilium movement and the acrosome reaction (Fig. 4B), implying predisposed expression of genes indispensable for fertilization. Furthermore, the relative expression levels of genes known to be essential for germ cell development also consistently fluctuated along with pseudotime (Fig. S4A). For example, genes promoting proliferation and differentiation of spermatogonia, such as *TOP2A*, *PIWL2*, *PIWL1* and *DMRT1* were highly expressed at spermatogonia and sharply downregulated at later stage (Fig. S4A). *RAD51AP2* and *SYCP* gene family encoding synaptonemal complex protein 1–3, required for synapsis, recombination, and segregation of meiotic chromosomes (Kovalenko et al., 2006), were highly expressed from spermatogonia to spermatocyte (Fig. S4A). *CATSPER* gene family (*CATSPER3* and *CATSPER4*) involved in flagellated sperm motility and sodium ion transport, and *SPACA1* responsible for acrosomal morphogenesis, were restricted to spermatozoa (Fig. S4A). Most interestingly, *PRM2*, which is responsible for compacting sperm DNA into a highly condensed and inactive complex, and *TNP2*, which is involved in the replacement of histones to protamine in the elongating spermatids, were persistently upregulated from elongating to elongated spermatid stages (Fig. S4A). Overall, these results demonstrate that pseudotime traced the sequential and stepwise developmental trajectory of male germ cells.

Next, to investigate age-associated alterations along the trajectories during spermatogenesis, we compared transcriptional profiles of module-specific genes between the young and the aged groups (Fig. 4C). Interestingly, we noticed downregulations of module 1-specific genes in aged spermatogonia, module 2-specific genes in aged Early SPC, module 3-specific genes in aged Late SPC, as well as module 4- and 5-specific genes in aged spermatid (Fig. 4C), suggesting dampened functions of corresponding spermatogenic stages. Consistently, the gene set scores for male gamete generation across all major cell types were much lower in aged cells than those in young cells (Fig. 4D and 4E). When we assessed the changes at each stage of spermatogenesis, we also observed a consistent dysregulation of each biological process in the aged testis, such as pronounced downregulation of genes responsible for mitosis (*TTN*, *KIF25*, *KIF2C*), meiosis (*BRCA2*, *BYN62*, *TEX15*), spermatid differentiation (*ODF2*, *PCSK4*, *TCP11*), as well as subsequent disrupted spermiogenesis (i.e., decreased genes related to cilium organization (*ODF2*, *CFAP100*, *AKAP4*), perinuclear theca (*SPATA3*, *SPATA6*, *CAPZA3*) and exacerbated abnormal sperm motility (*DNAH1*, *DNAH10*, *BICC1*)) (Figs. 4D, 4E, S4B and S4C).

Altogether, these observations pinpoint early molecular events in the aged germ cells, which may instigate the onset of overall impaired spermatogenesis.

### Attrition of spermatogonial stem cell reservoir in aged NHP testes

To gain a deeper insight into the age-accumulated molecular alterations in spermatogonia, we next focused on two distinct gene expression profiles of spermatogonial development (Fig. 4F and 4G). Genes in module 1 were progressively downregulated along the trajectory and highly expressed in SSC (Fig. 4G), and were closely correlated with regulation of the Wnt signaling pathway and cellular response to growth factor stimulus (Fig. 4G). By contrast, progressively upregulated genes converged in module 2 and were associated with mitotic cell cycle and DNA repair, which likely boosts mitosis in SSC and vigorous proliferation in Diff. SPG (Fig. 4G). Notably, we found an elevation of genes of module 2 emerged in aged SSC (Fig. 4H). These findings suggest loss of stemness maintenance along with a striking shift in transcriptional programs towards propagation with advanced age, accelerating the exhaustion of stem cell pool. Concurrently, the cell cycle phase analysis also uncovered an augmented proliferative S-phase in aged SSC, likely driving SSC exhaustion and overactive proliferation (Fig. 4I).

Subsequently, we performed single-cell regulatory network inference and clustering (SCENIC) analysis to identify transcription factors crucial for modulating cell fate of spermatogonia in each module (Fig. S4D). Interestingly, when we explored age-associated changes of these TFs, we found increased expression of *BRCA1* along the trajectory (Fig. 4D), over-expression of which disrupts the quiescence of hematopoietic stem and progenitor cell in the bone marrow (Bai et al., 2013), a mechanism that may also break SSC quiescence and accelerate attrition of the SSC pool. Meanwhile, blunted expression of stemness-associated TFs including *HMGA2* was present in the aged group, the depletion of which is known to impair neural stem cell stemness (Fig. S4D) (Nishino et al., 2008). These bidirectional changes may lay the foundation for depletion of SSC reservoir and consequent reduced spermatogonia in the aged NHP testes as we had observed (Fig. 2D and 2E). Consistently, canonical TFs for SSC maintenance, i.e., *TCF3* and *DMRT1*, also decreased along the pseudotime axis (Fig. S4D) (Zhang et al., 2016a).

In accordance with the aforementioned findings, we unraveled core TFs governing the aging-related DEGs in spermatogonia (Fig. S4E). Strikingly, *TCF7L1*, a stem cell regulator, progressively declined along the SPG pseudotime (Fig. S4E), whose blunted expression in the aged cells further supported loss of stemness maintenance in SSC during NHP aging. Similarly, the age-related elevated expression of *HMGB1*, which transactivates the cell senescence marker gene *p21*^Cip/WAF1^ and orchestrates STING-mediated senescence via modulation of TRIM30α expression (Lee et al., 2019, 2021), across the spermatogonia trajectory may incur exacerbated senescence and eventually impair spermatogonia pool (Fig. S4E). Taken together, these findings delineate age-related transcriptional signatures underpinning imbalance of self-renewal and differentiation in aged spermatogonia stem cells.

### Dysregulated Sertoli cells serve as a hostile microenvironment that compromises spermatogenesis in aged NHP testes

**Figure 5.**
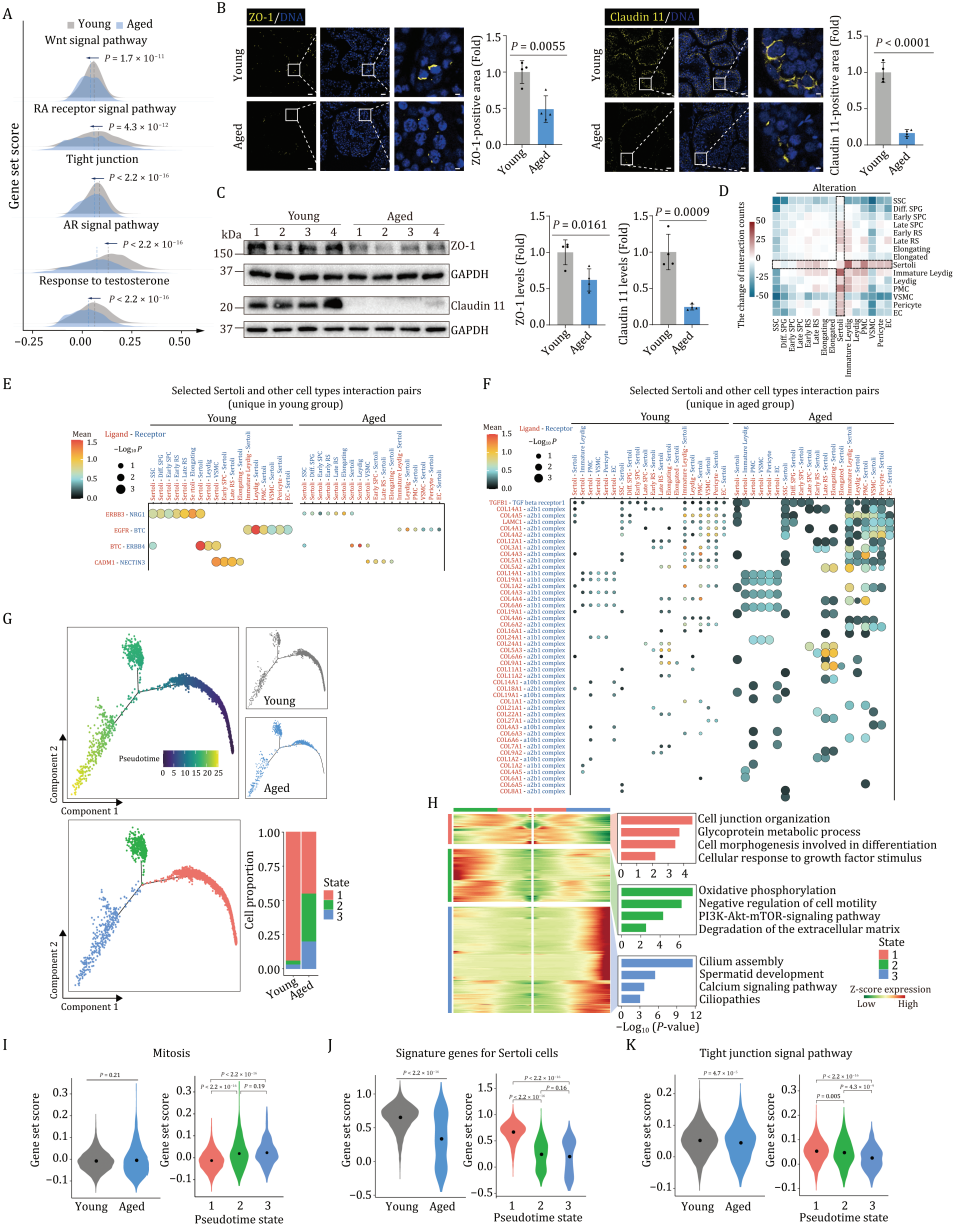
**Dysregulation of Sertoli cells is identified as a crucial contributor to hostile microenvironment in aged NHP testes.** (A) Ridge plots showing the representative gene set scores for Sertoli cells in young and aged groups. (B) Immunofluorescence analysis of ZO-1, Claudin 11 expression in testicular tissues from young and aged monkeys. Representative images are shown on the left. ZO-1 or Claudin 11-positive areas are quantified as fold changes (Aged vs. Young), and shown on the right. Young, *n* = 4; aged, *n* = 4 monkeys. Scale bars, Scale bars: 20 μm and 3.3 μm (zoomed-in image). (C) Western blot analysis of ZO-1, Claudin 11 protein expression in testicular tissues from young and aged monkeys. Left, representative images. Right, protein levels are quantified as fold changes (Aged vs. Young), and shown as means ± SEM. Young, *n* = 4; aged, *n* = 4 monkeys. (D) Heatmap showing the aging-related alteration of cell–cell interaction numbers across cell types in monkey testes. (E) Dot plot showing the selected interaction pairs unique in young group between Sertoli and other cell types in monkey testes. (F) Dot plot showing the selected interaction pairs unique in aged group betweenSertoli and other cell types in monkey testes. (G) Pseudotime analysis of Sertoli cells in monkey testes. Upper left, pseudotime scores of Sertoli cells in monkey testes. Upper right, the distribution of young and aged groups along the pseudotime trajectory. Lower left, the distribution of different Sertoli subtypes (named as state 1, state 2, and state 3) along the pseudotime trajectory. Lower right, bar plot showing the distribution of Sertoli subtype proportion between young and aged groups. (H) Expression profiles of top 500 DEGs and their enriched GO terms for different clusters along the pseudotime trajectory. Left, heatmaps showing the expression profiles of top 500 DEGs for different clusters along the pseudotime trajectory. Right, the enriched GO terms of genes in each cluster. (I) Violin plots showing the gene set scores for mitosis-related genes. Left, mitosis gene set scores in young and aged groups. Right, mitosis gene set scores for different states of Sertoli cells along the pseudotime axis. (J) Violin plots showing the gene set scores for the signature genes for Sertoli cells (top 50 marker genes of Sertoli cells ranked by LogFC). Left, Sertoli signature gene set scores in young and aged groups. Right, signature gene set scores for different states of Sertoli cells along the pseudotime axis. (K) Violin plots showing the gene set scores for genes involving in tight junction signal pathway. Left, tight junction signal pathway gene set scores in young and aged groups. Right, tight junction signal pathway gene set scores in different states of Sertoli cells along the pseudotime axis.

Sertoli cell is a key orchestrator of multiple testicular functions (Griswold, 1998; O’Donnell et al., 2022). Here, we found that Sertoli cell was the most responsive cell type to aging, as manifested by the highest number of aging-related DEGs and the highest transcriptional noise (Fig. 3A and 3B). We next focused on dissecting the underpinning of molecular events essential for age-associated Sertoli Cell dysfunction (Fig. S5A). The integrity of blood-testis barrier (BTB), consisting of inter-cellular tight junction among Sertoli cells, is critical for meiosis and post-meiotic spermatid development (Wong and Cheng, 2005). Intriguingly, we found a remarkable reduction of gene set score of genes encoding tight junction in the aged Sertoli cells (Fig. 5A). Consistently, key tight junction proteins (ZO-1, Claudin 11) exhibited decreased expression and discontinuous distribution along the cell membrane periphery (Fig. 5B and 5C), implying potential disruption of intact tight junction. In addition, we observed a sharp decrease of genes related to sex hormone signaling pathways pivotal for germ cell development (Griswold, 1998; Shah et al., 2021; O’Donnell et al., 2022), including response to testosterone and the AR signal pathway in aged NHP Sertoli cells (Fig. 5A). Other growth factor-related pathways crucial for Sertoli cell differentiation and germ cell development were also scored and downregulated in the aged Sertoli cells relative to those in young counterparts, such as the Wnt signal pathway and the RA receptor signal pathway (Fig. 5A). Overall, these findings reveal dysfunctional state in aged NHP Sertoli cells.

As the interactions between Sertoli cells and the germ cells play a central role in spermatogenesis (Griswold, 1995; Syed and Hecht, 2002), we next performed cell-cell interaction analysis and found a global dysregulation of cell-cell interactions between aged Sertoli cells and other testicular cell types (Figs. 5D–F, and S5B). Specifically, decreased ligand-receptor pairs between aged Sertoli cells and germ cells mainly included NRG1 signaling and cell–cell junction. Among them, the ERBB3_NRG1 interaction between Sertoli cell and germ cells was remarkably lost in the aged NHP testes (Fig. 5E), which may contribute to a depleted Sertoli cell stock and potential spermatogenesis failure (Gregoire et al., 2018). In addition, erased CADM1_NECTIN3 pair was observed between Sertoli cells and Early SPC/Late RS/Elongating spermatid (Fig. 5E). This finding is likely to dampen transportation of early spermatocytes, residing in the basal compartment, across the BTB to enter the adluminal compartment, thus compromising spermatid development (Inagaki et al., 2006; Maekawa et al., 2011). Conversely, increased ligand-receptor pairs between aged Sertoli cells and germ cells were enriched in fibrosis and inflammation-related damages (Fig. 5F). The representative pairs were TGFB1 and TGF beta receptor 1 as well as COL14A1 and integrin a1b1 complex (Fig. 5F), suggesting a destabilization of the supportive microenvironment, ultimately leading to spermatogenesis impairment (Stewart et al., 2018).

In pursuit to provide the detailed view of the effects of aging on Sertoli cells, we next re-clustered the Sertoli cells and found that they partitioned into three distinct states (Fig. 5G). When we analyzed the transcriptomic profiling across the states along the pseudotime axis (Fig. 5H), we found that genes in state 1 were functionally linked to cell junction organization and cell morphogenesis with high expression of *NRCAM* and *BCL2L2-PABPN1* (Figs. 5H and S5C). A panel of genes including *TSHZ2* and *GABRG3* marked state 2, and were associated with negative regulation of cell motility and degradation of the extracellular matrix (Figs. 5H and S5C). Genes in state 3 were enriched for Ciliopathies, marked by *NKAIN3* and *CNBD1* (Figs. 5H and S5C). To be noted, Sertoli cells in state 2 and state 3 were highly enriched with age, and expanded at the advanced age (Fig. 5G). In addition to potentially deleterious effects on cilium or flagellum-dependent sperm motility, we also noticed a wide range of dysregulated pathways crucial for Sertoli cell differentiation and maturation in state 2 and 3, including increased mitosis, as well as blunted cell identity signature, tight junction, response to sex hormone and growth factor-related pathways (Figs. 5I–K and S5D). Together, the results suggested loss of terminally differentiated state and accompanying profound dysfunction of Sertoli cells in the aged NHP testes.

### Silence of WT1 induces senescence of human Sertoli cells

**Figure 6.**
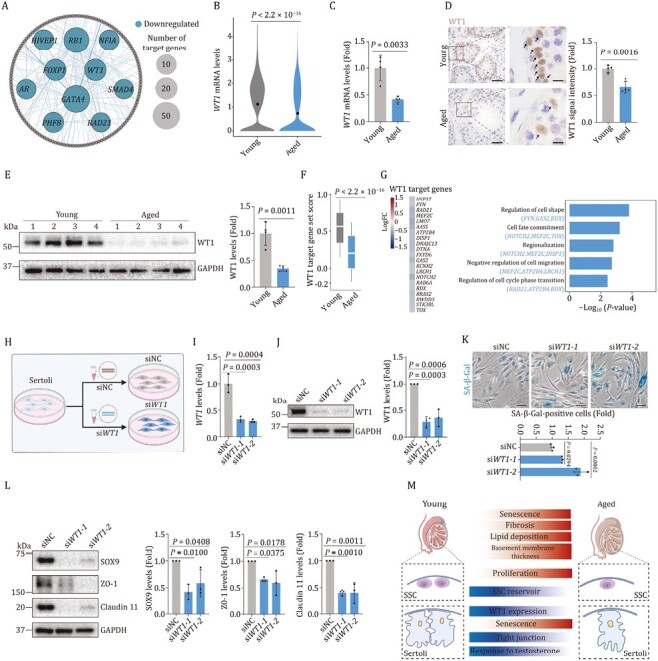
**Knockdown of *WT1* accelerates senescence of human Sertoli cells.** (A) Network plot showing the downregulated core TFs predicted by SCENIC using aging-related DEGs of Sertoli cells. Inter nodes represent downregulated core TFs and node size positively correlates with the number of target genes regulated by specific TF. Each outer node represents one target gene. (B) Violin plot showing the *WT1* mRNA expression levels in young and aged testes. (C) RT-qPCR analysis of *WT1* mRNA expression in testicular tissues from young and aged testes. *WT1* mRNA levels are quantified as fold changes (Aged vs. Young), and shown as means ± SEM on the right. Young, *n* = 4; aged, *n* =4 monkeys. (D) Immunohistochemistry analysis of WT1 in testicular tissues in young and aged testes. Representative images are shown on the left. WT1 signal intensity is quantified as fold changes (Aged vs. Young), and shown as means ± SEM on the right. Young, *n* = 4; aged, *n* = 4 monkeys. Scale bars, 50 μm and 12.5 μm (zoomed-in image). (E) Western blot analysis of WT1 protein expression in testicular tissues in young and aged testes. WT1 protein levels are quantified as fold changes (Aged vs. Young), and shown as means ± SEM on the right. Young, *n* = 4; aged, *n* = 4 monkeys. (F) Box plot showing the gene set scores of WT1 target genes predicted by SCENIC in young and aged testes. (G) The expression levels of WT1 target genes and their enriched GO terms in young and aged groups of Sertoli cells. Left, heatmap showing the expression levels of WT1 target genes. Right, bar plots showing the enriched GO terms of WT1 target genes. (H) Schematic diagram showing the procedure of *WT1*-knockdown assay in human Sertoli cells. (I) Validation of the knockdown efficiency by RT-qPCR. *WT1* mRNA levels are quantified as fold changes (si*WT1* vs. siNC), and shown as means ± SEM on the right. *n* = 3, three independent replicates. (J) Validation of the knockdown efficiency by Western blot. WT1 protein levels are quantified as fold changes (si*WT1* vs. siNC), and shown as means ± SEM on the right. *n* = 3, three independent replicates. (K) SA-β-Gal staining of human Sertoli cells upon knockdown of *WT1*. Representative images are shown on the left. SA-β-Gal positive cells are quantified as fold changes (si*WT1* vs. siNC), and shown as means ± SEM on the right. *n* = 3, three independent replicates. (L) Western blot analysis of SOX9, ZO-1 and Claudin 11 protein expression in Sertoli cells upon knockdown of *WT1*. Relative protein levels are quantified as fold changes (si*WT1* vs. siNC), and shown as means ± SEM on the right. *n* = 3, three independent replicates. (M) A schematic illustration showing the phenotypic and transcriptomic signatures of NHP testicular aging.

To further explore core TFs modulating aging-related DEGs in Sertoli cells, we constructed a transcriptional regulatory network through SCENIC analysis (Figs. 6A and S6A). Interestingly, the downregulated transcriptional regulators included a set of core TFs essential for Sertoli cell function, such as *WT1*, *GATA4* and *AR* (Figs. 6A and S6B). Amongst them, WT1 directs Sertoli cell lineage specification and maturation, and modulates cell polarity and tight junction (Wang et al., 2019). Indeed, *WT1* transcription was downregulated in aged Sertoli cells, especially in state 2 and state 3, along with a reduction in protein levels of WT1 in aged testes (Figs. 6B–E and S6C). Intriguingly, decreased WT1 expression was also observed in oxidative stress-induced senescent human Sertoli cells (Fig. S6D and S6E). When we further investigated the activity of the WT1 modulated regulatory network, we found that the score of predicted WT1-target genes was also lower in aged Sertoli cells relative to their young counterparts (Fig. 6F). The downregulated WT1-target genes were relevant to cell fate commitment (*MEF2C*, *NOTCH2*, *TOX*) and regulation of cell shape (*FYN*, *GAS2*, *RDX*) (Fig. 6G), processes important for maintaining Sertoli cell identity and stability in testicular cell junction. For instance, as FYN specifically localizes in ectoplasmic specializations of the Sertoli cells and regulates the dynamics of cytoskeletal proteins (Maekawa et al., 2002), downregulation of FYN likely impairs survival and differentiation of germ cells during spermatogenesis (Luo et al., 2012). These data suggest that age-related downregulation of WT1 and its downstream cascade may contribute to the dysregulated homeostasis of aged Sertoli cells and may underlie NHP testicular involution.

To experimentally validate the functional role of WT1 in mediating Sertoli cell senescence as we observed in aged NHP testes (Fig. 1G and 1H), we performed genetic perturbations of WT1 using either siRNA- or CRISPR/Cas9-mediated knockdown in human Sertoli cells and observed a massive reduction of WT1 expression at mRNA and protein levels (Figs. 6H, 6J and S6F). As expected, we found elevated SA-β-Gal-positive cells upon knockdown of *WT1* (Figs. 6K and S6G), indicating an accelerated senescence. Concomitantly, the expression levels of hub TFs crucial for maintaining Sertoli cell identity (e.g., SOX9) and several key tight junction proteins (e.g., ZO-1, Claudin 11) were diminished upon WT1 silencing in human Sertoli cells (Fig. 6L). Consistently, in addition to the age-related downregulation of WT1, we also observed an age-dependent downregulation of TF critical for Sertoli cell maintenance (e.g., SOX9) and tight junction protein (e.g., ZO-1) in aged mouse testes concomitantly, suggesting evolutionarily conservation of aging-related regulatory mechanism (Fig. S6H and S6I). Collectively, silencing of WT1 underpins dysregulations of primate Sertoli cells and may engender testicular dyshomeostasis and degeneration.

## Discussion

The testis executes spermatogenesis and steroidogenesis that support the functions of male reproductive system, and its age-associated functional decline debilitates fertility and increases reproductive diseases risk. However, to date, a comprehensive molecular survey of primate testicular aging has been lacking. Here, for the first time, we established a single-nucleus transcriptomic landscape of NHP testicular aging and analyzed the transcriptomic profile alterations along the spermatogenesis continuum and in the somatic cells that constitute the spermatogenic milieu (Fig. 6M). Our work provides a rich resource for mining the deeper intrinsic and extrinsic molecular events underlying primate testicular aging and paves the way for identifying potential biomarkers and targets for earlier clinical diagnosis and development of potential therapeutic interventions to treat aging-related testicular disorders.

Although aging of the male reproductive system is mild to moderate with age in men, paternal aging is a one of major risk factors for decline in reproductive outcomes (Wiener-Megnazi et al., 2012). Therefore, exploration of earlier intrinsic and extrinsic molecular events predisposing spermatogenesis failure is important to develop strategies that prevent progression to infertility. In the present study, we unraveled the transcriptional profiles underlying premature depletion of the spermatogonial stem cell reservoir, disturbed meiosis and impaired spermiogenesis within the sequential continuum. Amongst the cellular events, exhaustion of the spermatogonial stem cell reservoir, i.e. the cell fate shift from a well-maintained stemness towards accelerated attrition in aged SSCs, may serve as a central driver for testicular aging and potentially deteriorated spermatogenesis (Griswold, 1998; Oatley and Brinster, 2006; Komeya and Ogawa, 2015; Kubota and Brinster, 2018). Although the regulatory mechanism remains largely unresolved, emerging evidence points out aberrant asymmetric division, i.e., overactive division biased towards the differentiated daughter cell at the expense of the quiescent stem cell, may explain age-dependent stem cell exhaustion (Florian and Geiger, 2010; Inaba and Yamashita, 2012; Chhabra and Booth, 2021). In supporting our findings, aberrant asymmetric division has been reported to contribute to cellular and tissue aging in a variety of adult stem cells (Florian and Geiger, 2010; Goodell and Rando, 2015; Urban et al., 2019). Overall, our data establish cell-intrinsic transcriptomic fingerprints that carry a risk for spermatogenesis failure and underscore exhaustion of spermatogonial stem cells as a hallmark feature of NHP testicular aging.

It has been reported that dysregulation of testicular somatic cells contributes to a hostile milieu that impairs testicular function (Alfano et al., 2021; Wang et al., 2022b). Among these, Sertoli cell, also named as the testicular “nurse cell”, ranks as a key niche cell that supports the germ cell development (O’Donnell et al., 2022). In aged Sertoli cells, we detected the highest transcriptional variation and the greatest number of aging-related DEGs, suggesting Sertoli cells were the most responsive to changes caused by aging. When we mapped the transcriptional profiling to functional pathways, we found a remarkable reduction of genes encoding tight junction components in the aged Sertoli cells. As such, these results suggest a disruption of the blood-testis barrier (BTB), a central feature of robust spermatogenesis maintenance (Wong and Cheng, 2005). Moreover, decreased AR, signifying a compromised sex hormone response, was one of the downregulated TFs in aged NHP Sertoli cells. In supporting our observations, a pronounced decrease in testosterone levels is considered as a hallmark feature in the aged men, and the lack of AR in Sertoli cells segregates with infertility (Zirkin and Tenover, 2012; Cao et al., 2021). Overall, the identification of cellular and molecular events predisposing to spermatogenesis failure deepens our understanding of progressive degeneration in the primate testis.

Intriguingly, we identified that WT1 downregulation in the aged testis serves as a potential driving force triggering accelerated senescence in Sertoli cells and may engender subsequent testicular involution. WT1 is known to play multiple roles in development, tissue homeostasis and disease (Hastie, 2017). In mouse testis development, WT1 has been reported to inhibit aberrant beta-catenin signaling in Sertoli cells, and deletion of WT1 causes changes in Sertoli cell identity and misregulation of inter-Sertoli cell contacts (Chang et al., 2008). Another study demonstrated that WT1 is essential for maintaining the integrity of testicular cords by regulating the expression of Col4a1 and Col4a2 (Chen et al., 2013). In the adult testis, WT1 maintains the Sertoli cell lineage and ablation of WT1 leads to the reprogramming of Sertoli cells to Leydig cells (Zhang et al., 2015). However, the function of WT1 in response to aging, especially in primates, has remained elusive. Herein, we first demonstrated an unappreciated geroprotective role of WT1 in NHP Sertoli cells at least partially by modulating tight junction stability and Sertoli cell identity. Since ZO-1 and several claudin proteins have been reported to be degraded through autophagy-lysosome pathway (Liu et al., 2016; Zhang et al., 2018; Ganapathy et al., 2022), and WT1 was involved in the Akt-JNK pathway dependent autophagy in human osteosarcoma cells (Mo et al., 2016), we speculate that WT1 may regulate the expression of tight junction proteins (ZO-1 and Claudin 11) through autophagy-lysosome pathway, which needs further investigation. In addition, given a previous study showing that SOX9 expression declines rapidly after WT1 deletion in mice (Chen et al., 2013), we infer that WT1-silencing in primate Sertoli cells may also result in a cell fate shift, which in turn causes age-associated loss of functional Sertoli cells and consequent testicular degeneration. Thus, our finding extends the biological implications of WT1 in the testicular homeostasis during aging, and offers novel therapeutic targets for intervening in NHP testicular aging and related diseases.

In sum, we construct a comprehensive single-nucleus transcriptomic atlas of primate testicular aging that deepens our understanding of diverse mechanisms underlying testicular aging in primates. Our work serves as a rich resource for identifying potential biomarkers for earlier clinical diagnosis of testicular aging and targets for development of novel therapeutic interventions to treat aging-related testicular disorders.

## Materials and methods

### Testicular tissue collection

Testicular tissues from four young (5–6 years old) and four aged (18–21 years old) male cynomolgus monkeys (*Macaca fascicularis*) were collected as previously described (Ma et al., 2020; Zhang et al., 2020, 2021; Li et al., 2021; Wang et al., 2021b). After perfusion with 0.9% saline, we carefully picked out the right testis on ice, sliced them into pieces orderly, put them in several freezing tubes or embedded with optimal cutting temperature compound (O.C.T) (Sakura, 4583), and stored them in liquid nitrogen. Meanwhile, the left testis of each monkey was cut in half and fixed in 4% paraformaldehyde for paraffin embedding and histological analyses.

### SA-β-Gal staining

SA-β-Gal staining was performed following the previously published protocols (Debacq-Chainiaux et al., 2009; Wang et al., 2018; Ma et al., 2020, 2021). For cells, the cultured Sertoli cells at a suitable confluent were washed with PBS twice, fixed in fixation buffer (2% formaldehyde and 0.2% glutaraldehyde) at room temperature (RT) for 5 min, washed with PBS twice and stained with freshly prepared SA-β-Gal staining solution (5 mmol/L K_4_[Fe(CN)_6_], 5 mmol/L K_3_[Fe(CN)_6_], 150 mmol/L NaCl, 2 mmol/L MgCl_2_, 40 mmol/L citric acid/Na phosphate buffer, 1 mg/mL X-gal (Amresco, 0428)) at 37°C for 20 h. For tissues, in brief, the O.C.T-embedded testicular tissues were cryosectioned at a thickness of 10 μm with a Leica CM3050S cryomicrotome, mounted on Superfrost Plus microslides (VWR) and stored at −80°C. Before SA-β-Gal staining, sections were thawed at RT and rinsed in PBS for 5 min, fixed in fixation buffer at RT for 5 min and stained with freshly prepared SA-β-Gal staining solution at 37°C for 16 h. Images were taken with Olympus CKX41 microscope imaging system, and the SA-β-Gal-positive areas were quantified with ImageJ.

### SPiDER-βGal staining

SA-β-Gal activity was stained with SPiDER-βGal (Dojindo­Molecular Technologies, Inc., Kumamoto, Japan) according to the manufacturer’s instruction. In brief, the O.C.T-embedded testicular tissues were cryosectioned at a thickness of 10 μm with a Leica CM3050S cryomicrotome, mounted on Superfrost Plus microslides (VWR) and stored at −80°C. Then, the slides were fixed in 4% paraformaldehyde for 20 min at RT, washed in PBS, and immersed in 20 μmol/L SPiDER-βGal staining working solution (Dojindo Molecular Technologies, SG03, 1:2,000) diluted with McIlvaine buffer (pH 6.0) for overnight at 4°C. Then, the sections were counterstained with Hoechst 33342 (Thermo Fisher, H3570, 1:1000), washed with PBS and mounted in VECTERSHIELD^®^ anti-fading mounting medium (Neobioscience, H-1000). Zeiss LSM900 confocal system was used for the image capture.

### Immunofluorescence staining

Immunofluorescence staining was conducted as previously described (Li et al., 2021; Ma et al., 2021; Wang et al., 2022a). Briefly, O.C.T-embedded testicular tissue sections with a 10 μm thickness were rinsed in distilled water, permeabilized with 0.4% Triton X-100 for 30 min and washed again in PBS for three times. Then, sections were incubated with blocking buffer (10% donkey serum in PBS) at RT for 1 h, primary antibodies overnight at 4°C and fluorescence-labeled secondary antibodies at RT for 1 h. The sections were counterstained with Hoechst 33342 (Thermo Fisher, H3570, 1:1000) to visualize the nuclei. Finally, the sections were mounted in VECTERSHIELD^®^ anti-fading mounting medium (Neobioscience, H-1000). Zeiss LSM900 confocal system was used for the image capture. The primary antibodies used for immunofluorescence staining in this study are as follows: anti-Collagen IV (Millipore, AB769, 1:200), anti-WT1 (abcam, ab89901, 1:200), anti-UTF1 (Millipore, MAB4337, 1:1000), anti-SMA (abcam, ab150301, 1:200), anti-ZO-1 (Invitrogen, 617300, 1:200), anti-Claudin 11 (Invitrogen, 364500, 1:200). Secondary antibodies used are as follows: donkey anti-mouse-AF488 (Thermo Fisher, A21202, 1:500), donkey anti-rabbit-AF568 (Thermo Fisher, A10042, 1:500), donkey anti goat-AF647 (Thermo Fisher, A21447, 1:500). In this study, immunofluorescence staining results were quantified by two ways, including the proportion of positive cells and the ratio of positive areas, and the specific statistical methods were performed according to previous published studies (Basu et al., 2018; Cansby et al., 2018; Schwayer et al., 2019).

### SA-β-Gal/SPiDER-βGal and immunofluorescence co-staining

For SA-β-Gal/SPiDER-βGal and immunofluorescence co-staining, after the SA-β-Gal/SPiDER-βGal staining procedure, the sections were washed with PBS, then fixed with 4% paraformaldehyde fix solution for 20 min, washed twice with PBS, permeabilized with 0.4% Triton X-100 for 1 h and rinsed again in PBS three times. Sections were then incubated with blocking buffer (10% donkey serum in PBS) at RT for 1 h, followed by incubation with primary antibodies overnight at 4°C and fluorescence-labeled secondary antibodies at RT for 1 h. Nuclei were counterstained with Hoechst 33342 (Thermo Fisher, H3570, 1:1000) before the sections were mounted in VECTERSHIELD^®^ anti-fading mounting medium (Neobioscience, H-1000). Zeiss LSM900 confocal system was used for the image capture. The antibodies used for immunofluorescence co-staining with SA-β-Gal/SPiDER-βGAL are as follows: anti-Collagen IV (Millipore, AB769, 1:200), anti-WT1 (abcam, ab89901, 1:200), anti-UTF1 (Millipore, MAB4337, 1:1000), Secondary antibodies used are as follows: donkey anti-mouse-AF488 (Thermo Fisher, A21202, 1:500), donkey anti-rabbit-AF568 (Thermo Fisher, A10042, 1:500), donkey anti goat-AF647 (Thermo Fisher, A21447, 1:500).

### Masson-trichrome staining

In brief, paraffin-embedded testicular tissues sections with a 5 μm thickness were first deparaffinized in xylene and then rehydrated in a graded series of alcohols (100%, 100%, 95%, 80%, 75%) and washed in distilled water. Then sections were stained with potassium dichromate solution at 60°C for 1 h and washed in running water for 10 min. Then, the sections were incubated sequentially in Weigert’s iron hematoxylin working solution for 3 min, in Biebrich scarlet-acid fuchsin solution for 10 min, and in phosphomolybdic-phosphotungstic acid solution for 10 min. Between each step, the slides were washed in distilled water. Then the sections were transferred directly to aniline blue solution and stained for 5 min. After washing briefly in distilled water, the sections were differentiated in 1% acetic acid solution for 2 min and rinsed in distilled water. At last, the sections were dehydrated and mounted with resinous mounting medium. Images were taken with PerkinElmer Vectra Polaris.

### Lipofuscin staining by Sudan black B (SBB)

Sudan black B staining was performed as previously described (Georgakopoulou et al., 2013). In brief, O.C.T-embedded testicular tissue sections with a 10 μm thickness were fixed in 1% (*w*/*v*) formaldehyde/PBS for 5 min at RT and then washed 1 min for three times with PBS. Sections were then incubated for 5 min in 50% ethanol and then for another 5 min in 70% ethanol. Then, sections were incubated with SSB staining solution (0.7g SBB in 100 mL 70% ethanol) for 5 min. The sections were rinsed in 70% ethanol and the staining was observed under the microscope. The tissues were then rinsed into 50% ethanol, washed in distilled water, stained with 0.1% Nuclear Fast Red (Beyotime, C0151) for 2 min, and mounted into 50% Glycerol/TBS mounting medium. The Blue-black granules aggregates in perinuclear and cytoplasmic area are considered to be positive signal for lipofuscin staining. Images were taken with Olympus CKX41 microscope imaging system, and the lipofuscin-positive areas were quantified with ImageJ.

### Oil Red O staining

Oil Red O staining was conducted as previously described (Khawar et al., 2021). Briefly, O.C.T-embedded testicular tissue sections with a 10 μm thickness were fixed with 4% paraformaldehyde for 20 min followed by washing three times with PBS. Then, sections were rinsed in 60% (*v*/*v*) isopropyl alcohol for 5 min and air-dried. Next, the air-dried sections were stained for 15 min using 60% Oil Red O solution. To wipe off the background staining, the slides were washed with 70% ethanol for 5 s. Furtherly, the slides were rinsed and counterstained with Harris hematoxylin and mounted with 50% glycerol/PBS. Images were taken with Olympus CKX41 microscope imaging system, and the Oil Red O-positive areas were quantified with ImageJ.

### TUNEL staining

To identify the apoptotic signals within testicular tissues, the TUNEL staining was performed using the One Step TUNEL Apoptosis Assay Kit (Beyotime, C1088) following the manufacturer’s instruction. In brief, paraffin-embedded testicular tissue sections with a 5 μm thickness were routinely dewaxed to water. The sections were incubated with proteinase K (dilution with 10 mmol/L Tris-HCl, 1:1000) for 30 min at RT, then rinsed in PBS. Next, the sections were stained with TUNEL working solution at 37°C for 1 h. Then the slides were counterstained with Hoechst 33342 (Thermo Fisher, H3570, 1:1000) to visualize the nucleus. Finally, the slides were mounted with VECTERSHIELD^®^ anti-fading mounting medium (Neobioscience, H-1000). Zeiss LSM900 confocal system was used for the image capture.

### Immunohistochemistry staining

Immunohistochemistry staining was performed as previously described (Li et al., 2021; Ma et al., 2021). In brief, the paraffin-embedded testicular tissues sections with a 5 μm thickness were first deparaffinized in xylene and then rehydrated in a graded series of alcohols (100%, 100%, 95%, 80%, 75%) and washed in distilled water. Sections were subjected to antigen retrieval by boiling in citrate buffer five times for 5 min each and cooled down to RT. Then, sections were rinsed in PBS, permeabilized with 0.4% Triton X-100 in PBS for 30 min and rinsed again in PBS for three times. To inactivate endogenous peroxidase, the sections were incubated with 3% H_2_O_2_ for 15 min at RT. Sections were then incubated with primary antibodies (dilution with 5% donkey serum in PBS) at 4°C overnight. The second day, sections were incubated with HRP-conjugated secondary antibodies for 1 h at RT. Sections were conducted with the DAB Staining Kit (ZSGB-BIO, ZLI-9018) according to the manufacturer’s protocols. Finally, sections were routinely dehydrated in a series of graded alcohols and xylene, mounted with the neutral resinous mounting medium. Images were taken with PerkinElmer Vectra Polaris. The antibodies used for immunohistochemistry staining in this study are listed as follows: anti-Collagen IV (abcam, ab6586, 1:200), anti-WT1 (abcam, ab89901, 1:200), anti-UTF1 (Millipore, MAB4337, 1:1000), anti-SOX9 (Millipore, AB5535, 1:1000), anti-INSL3 (Novus biological, NBP1-81223, 1:500), anti-c-KIT (abcam, ab32363, 1:200).

### Cell culture and transfection

The human immortal Sertoli cell line (MZ-4023, Mingzhoubio) is purchased from Mingzhou Biotechnology Co., Ltd (Ningbo, China) and cultured in complete medium for human testicular Sertoli cells (CM-H060, Mingzhoubio) supplemented with testicular Sertoli cell growth factor (254726, Mingzhoubio). siRNAs were synthesized by Tsingke Biotechnology Co., Ltd (Beijing, China) with sequences as follows si*WT1*-1: 5ʹ-ACAACAUGCAUCAGAGAAA-3ʹ; si*WT1*-2: 5ʹ-GUGGCCAAGUUGUCAGAAA-3ʹ. For siRNA transfection, Lipofectamine^®^ RNAiMAX Reagent (Invitrogen, 13778150) was used for the transfections of RNA oligonucleotides as the manufacturer’s instructions (Invitrogen, Carlsbad, CA).

### CRISPR/Cas9-mediated *WT1* knockdown in Sertoli cells

Guide RNAs (gRNAs) of *WT1* were designed using optimized CRISPR design by GenCRISPR gRNA design tool. *WT1* sgRNAs were listed as follows: sg*WT1-1*: 5ʹ-GGGCTCGCTGGGTAAGCGAG-3ʹ; sg*WT1*-2: 5ʹ-GAGTAGCCCCGACTCTTGTA-3ʹ, and were cloned into lenti-CRISPR v2 puromycin plasmid. For lentivirus generation, HEK293T cells were co-transfected with WT1 lentiviral vectors, psPAX2 (Addgene, #12260) and pMD2.G (Addgene, #12259) using PEI MAX^®^—Transfection Grade Linear Polyethylenimine Hydrochloride (MW 40,000) (Polysciences, 24765). The Virus supernatant were collected at 48 h and 72 h after transfection and then subjected to ultracentrifugation for viral concentration. Sertoli cells at optimal confluence were infected with suitable titers of sg*WT1* or sg-Negative Control (sg*NC*) lentivirus in the presence of polybrene (5 μg/mL) and positive cell pools were selected with puromycin (1 μg/mL). The knockdown efficiency was validated by Western blot analysis.

### Western blot

Testicular tissues or Sertoli cells were lysed with RIPA lysis buffer (50 mmol/L Tris-HCl at pH 7.4, 150 mmol/L NaCl, 1% NP-40, 1% sodium deoxycholate, 0.1% SDS) with protease inhibitor cocktail. Cell lysates were quantified by the Pierce™ BCA Protein Assay Kit (Thermo Fisher, 23225) and heated at 100°C in SDS-loading buffer for 10 min. The samples were subjected to SDS-PAGE and electrotransferred to PVDF membranes (Millipore). Then the membrane was blocked with 5% non-fat milk, and incubated with primary antibodies for overnight at 4°C. Next day, the membrane was incubated with HRP-conjugated secondary antibodies. Signals were captured by the ChemiDoc XRS system (Bio-Rad). Antibodies used for Western blot analysis in this study are as follows: anti-WT1 (abcam, ab89901, 1:1000), anti-SOX9 (Millipore, AB5535, 1:2,000), anti-ZO-1 (Invitrogen, 617300, 1:500), anti-Claudin 11 (Invitrogen, 364500, 1:1000), anti-GATA-4 (Santa Cruz, sc-9053, 1:1000), anti-GAPDH (Santa Cruz, sc-365062, 1:4,000), goat anti-Rabbit IgG (ZSGB-bio, ZB-2307, 1:5,000), goat anti-Mouse IgG (ZSGB-bio, ZB-2305, 1:500).

### Quantitative reverse transcription PCR (RT-qPCR)

For RT-qPCR, RNAs from testicular tissues and Sertoli cells were isolated with Trizol (Invitrogen, 15596018) following the manufacturer’s instructions (Invitrogen, Carlsbad, CA). The cDNAs were synthesized by use of HiScript II Q Select RT SuperMix for qPCR (+gDNA wiper) (Vazyme, R233-01). Quantitative real-time PCR following reverse transcription was conducted with THUNDERBIRD SYBR^®^ qPCR Mix (TOYOBO, QPS-201C) on Bio-Rad CFX Opus 384 Real-Time PCR System. The RT-qPCR primers used in this study are as follows. *GAPDH* forward: 5ʹ-CTTGCGTATTCCCCCAGGTT-3ʹ; *GAPDH* reverse: 5ʹ-TTCCCGTTCTCAGCCTTCAC-3ʹ; *WT1* forward: 5ʹ-CTTAAAGGGAGTTGCTGCTGG-3ʹ; *WT1* reverse: 5ʹ-TGGTTATCGCTCTCGTACCC-3ʹ.

### Nuclei isolation and snRNA-seq for testicular tissues

Isolation of nuclei from frozen testicular tissues was performed as previously described (Krishnaswami et al., 2016; Ma et al., 2020; Zhang et al., 2021, 2022). First, preparation of reagents including Nuclei isolation media 1 (NIM1) (250 mmol/L sucrose, 25 mmol/L KCl, 5 mmol/L MgCl_2_, 10 mmol/L Tris buffer pH 8.0), Nuclei isolation media 2 (NIM2) is based on NIM1 with the addition of 1 μmol/L DTT and 1× protease inhibitor. Homogenization buffer was diluted with NIM2 containing 0.4 U/μL RNaseIn, 0.2 U/µL Superasin, 0.1% Triton X-100, 1 μmol/L propidium iodide (PI), and Hoechst 33342 (1:1000). All procedures were carried out on ice or at 4°C. Frozen testicular tissues (*n* = 8) were ground into powder and rinsed in 1 mL prechilled homogenization buffer. Next, the tubes were put into the freezing multisample tissue grinding system with a grind for 60 Hz, 30 s, 3 times. After the homogenization, the homogenate was filtered with a 40-μm cell strainer (BD Falcon) to collect the supernatant. Then, the nuclei were harvested by centrifugation at 2,500 ×*g* for 8 min at 4°C. Then, the precipitated nuclei were resuspended in PBS supplemented with 0.5% BSA, 0.2 U/μL RNaseIn. The Hoechst 33342 and PI double positive nuclei were sorted by fluorescence-activated cell sorting (FACS) (BD Influx) and counted with a dual-fluorescence cell counter (Luna-FLTM, Logos Biosystems). Mononuclear capture was performed with a 10× Genomics single-cell 3ʹ system. Approximately 7,500 nuclei were captured for each sample following the standard 10× capture and library construction (10× Genomics) and then sequenced on a NovaSeq 6000 sequencing platform (Illumina, 20012866).

### Pre-processing and quality control of snRNA-seq data

Raw data generated by the NovaSeq sequencing platform were first processed using bcl2fastq (version 2.20.0.422) to convert BCL files into FASTQ format. Next, the FASTQ reads were aligned to the pre-mRNA reference (Ensemble, Macaca_fascicularis_5.0) and counted using Cell Ranger (version 4.0.0) (10× Genomics) with the default parameters. CellBender (version 0.2.0) (Fleming et al., 2019) was applied to the count matrix of each sample to remove the contamination of background mRNA. The filtered matrixes were further analyzed with Seurat (version 3.2.2) (Butler et al., 2018). Nuclei with genes below 200 or mitochondrial genes ratio greater than 5% were discarded. DoubletFinder (version 2.0.3) (McGinnis et al., 2019) was used to detect and remove doublets. After integration and clustering, clusters lacking specific marker genes, dominating in one sample, and with relatively low gene content or high mitochondrial ratio were also discarded.

### Sample integration, clustering, and identification of cell types

Data normalization, integration, dimensionality reduction, clustering, and differential gene expression analysis were performed under the corresponding pipeline of Seurat package. The filtered count matrix of each sample was normalized using the “SCTransform” function. Features and anchors for downstream sample integration were selected using the “PrepSCTIntegration” and “FindIntegrationAnchors” functions, ensuring that the batch effect was effectively eliminated. After data integration and scaling, principal component analysis (PCA) was performed with the “RunPCA” function. The first 26 PCs were used to execute the dimensionality reduction with the ‘‘RunUMAP’’ and ‘‘FindNeighbors’’ functions. Clustering was performed using the “FindClusters” function with the resolution of 2.0. Next, cell types were identified according to the expression levels of the canonical marker genes in germ and somatic cells. After cell type identification, the marker genes of each cell type were calculated using the “FindAllMarkers” function with the cutoff of LogFC > 1 and adjusted *P*-values < 0.05 using Wilcoxon Rank Sum test. Marker genes for each cell type are shown in Table S1.

### Transcriptional noise analysis

Calculation of age-related transcriptional noise was used to estimate the aging effects on different cell types according to the pipeline of previous work (Angelidis et al., 2019). For each cell type with at least 10 young and aged cells, the transcriptional noise was quantified in the following steps. First, the total unique molecular identifier (UMI) of all cells was down-sampled so that all cells had equal library size. Next, the cell numbers were down-sampled to obtain the equal number of young and aged cells. The Euclidean distance between each cell and the corresponding cell-type mean within each age group was then calculated, which represented the transcriptional noise. Furthermore, the Euclidean distance for each monkey and the transcriptional noise ratios of the aged group compared to the young group were also calculated to indicate the change of transcriptional noise across samples and cells during aging.

### Calculation of aging-related differentially expressed genes

Differentially expressed genes (DEGs) in the aged group compared to the young group in monkey testes were analyzed by the “FindMarkers” function of Seurat using Wilcoxon Rank Sum test, and were identified with the cutoff of |LogFC| > 0.25 and adjusted *P*-values < 0.05. DEG lists for each cell type are shown in Table S2.

### GO term analysis

Metascape was used to perform GO enrichment analysis (version 3.5) (Zhou et al., 2019). Representative terms were selected with the cutoff of *P*-values < 0.01 and were then visualized with ggplot2 R package (version 3.3.2) (Wickham, 2016).

### Analysis of transcriptional regulatory network

The construction of transcriptional regulatory network was performed using SCENIC (version 1.1.2.2) (Aibar et al., 2017) workflow with default parameters. Transcription factors (TFs) of hg19 were downloaded as reference TFs via RcisTarget package (version 1.6.0). The inference of co-expression modules between TFs and the potential target genes was carried out by GRNBoost (Moerman et al., 2019) in python3.0 using the input gene expression matrix. For each co-expression module, the enriched binding motifs among all potential target genes were analyzed by RcisTarget. Only genes enriched the binding motifs of the corresponding TFs were identified as target genes and only target genes with high-confidence annotation were engaged in downstream analysis. Finally, visualization of transcriptional regulatory network was performed by Cytoscape software (version 3.8.0) (Shannon et al., 2003). TFs and potential target genes are shown in Table S3.

### Gene set score analysis

The canonical pathway was obtained from KEGG (Kanehisa et al., 2021) and MSigDB (Liberzon et al., 2015) database. Aging-related gene set was obtained from Aging Atlas (Aging Atlas, 2021). Disease-related gene sets were obtained from DisGeNET database (Piñero et al., 2020). WT1 target gene set was obtained from the SCENIC analysis described in the previous section. These gene sets were then used for scoring each input cell with the “AddModuleScore” function of Seurat based on gene expression level. Alternations in the scores of the aged group compared to the young group were analyzed via ggpubr R package (version 0.2.4) using Wilcoxon Rank Sum test. Gene sets used in this study are shown in Table S4.

### Pseudotime analysis

Pseudotime analysis was performed on germ (SSC, Diff. SPG, Early SPC, Late SPC, Early RS, Late RS, Elongating and Elongated) cells and SPG or Sertoli cells solely with the Monocle2 R package (version 2.14.0) (Trapnell et al., 2014). For the analysis of germ cells, the cell number was first down-sampled to 20,000. Top 200–2,000 highly variable genes (200 for SPG cells, 1,000 for Sertoli cells and 2,000 for germ cells) from the “SCTransform” and “FindAllMarkers” functions of Seurat package were used as ordering genes. The trajectories were plotted in two-dimensional space using the DDRTree dimensionality reduction algorithm and cells described above were ordered in pseudotime. DEGs along the pseudotime trajectory were identified by the “differentialGeneTest” and “BEAM_rest” functions of Monocle2 with the cutoff of *q*-value < 0.01. The top 1,000 DEGs along the pseudotime trajectory of germ cells were selected as *q* value = 0 and in descending order of gene expressed in the number of cells. The top 400 DEGs along the pseudotime trajectory of SPG cells and the top 500 DEGs along the pseudotime trajectory of Sertoli cells were selected in ascending order of q value. The top DEGs along the pseudotime trajectories are shown in Table S5.

### Cell–cell communication analysis

Cell–cell communication analysis was performed using the CellPhoneDB software (version 2.1.7) (Efremova et al., 2020). Only receptors and ligands expressed in more than 10% cells of any cell type from either young or aged groups were further evaluated. And only ligand-receptor pairs with a *P*-value < 0.01 were considered as valid cell-cell communication between any two cell types. Cell-cell interaction pairs lost and gained with age between Sertoli cells and other cell types are shown in Table S6.

### Statistical analysis

All experimental data were statistically analyzed using PRISM software (GraphPad 8 Software). Results were presented as mean ± SEM. Comparisons were conducted using the two-tailed Student’s *t*-test or one-sided or two-sided Wilcoxon Rank Sum test as indicated in figure legends. *P*-values are presented in indicated figures.

## Supplementary Material

pwac057_suppl_Supplementary_Materials

pwac057_suppl_Supplementary_Table_S1

pwac057_suppl_Supplementary_Table_S2

pwac057_suppl_Supplementary_Table_S3

pwac057_suppl_Supplementary_Table_S4

pwac057_suppl_Supplementary_Table_S5

pwac057_suppl_Supplementary_Table_S6

## Data Availability

The single-nuclei transcriptomic high-throughput sequencing data in this study have been deposited in Genome Sequence Archive (GSA) with the accession number of CRA007812. The data can also be visualized and accessed via an interactive user-friendly webtool of Aging Atlas. Other data or materials generated in this study are available from the corresponding authors upon reasonable request.

## Abbreviations

BM: basement membrane
CRISPR/Cas9: clustered regularly interspaced short palindromic repeats/CRISPR-associated protein 9
CV: coefficient of variation
DEGs: differentially expressed genes
diff. SPG: differentiated spermatogonia
Early RS: early round spermatid
EC: endothelial cells
ECM: extracellular matrix
FACS: fluorescence-activated cell sorting
late RS: late round spermatid
LOH: late-onset hypogonadism
NHP: non-human primate
NIM1: nuclei isolation media 1
NIM2: nuclei isolation media 2
PBS: phosphate buffer solution
PCA: principal component analysis
PI: propidium iodide
PMC: peritubular myoid cell
RT: room temperature
RT-qPCR: quantitative reverse transcription polymerase chain reaction
SA-β-Gal: senescence associated-β-galactosidase
SBB: sudan black B
SCENIC: single-cell regulatory network inference and clustering
scRNA-seq: single-cell RNA sequencing
sgNC: sg-negative control
siRNA: small interfering RNA
snRNA-seq: single-nucleus RNA sequencing
SPC: spermatocyte
SPG: spermatogonium
SSC: spermatogonial stem cell
TFs: transcription factors
TUNEL: TdT (terminal deoxynucleotidyl transferase)-mediated dUTP nick-end labeling
UMAP: uniform manifold approximation and projection
UMI: unique molecular identifier
VSMC: vascular smooth muscle cells

## Code availability

All the custom codes are available from the corresponding authors upon reasonable request.
