# Correction to: A single-nucleus transcriptomic atlas of primate testicular aging reveals exhaustion of the spermatogonial stem cell reservoir and loss of Sertoli cell homeostasis

**DOI:** 10.1093/procel/pwaf033

**Published:** 2025-05-20

This is a correction to: Daoyuan Huang, Yuesheng Zuo, Chen Zhang, Guoqiang Sun, Ying Jing, Jinghui Lei, Shuai Ma, Shuhui Sun, Huifen Lu, Yusheng Cai, Weiqi Zhang, Fei Gao, Andy Peng Xiang, Juan Carlos Izpisua Belmonte, Guang-Hui Liu, Jing Qu, Si Wang, A single-nucleus transcriptomic atlas of primate testicular aging reveals exhaustion of the spermatogonial stem cell reservoir and loss of Sertoli cell homeostasis, *Protein & Cell*, Volume 14, Issue 12, December 2023, Pages 888–907, https://doi.org/10.1093/procel/pwac057.

In a recent review of this article, the authors discovered an inadvertent duplication of a blot band in Fig. 6L due to a file version mix-up during proofing. The corrected figure is shown below. This correction does not impact the study’s conclusions or discussion.


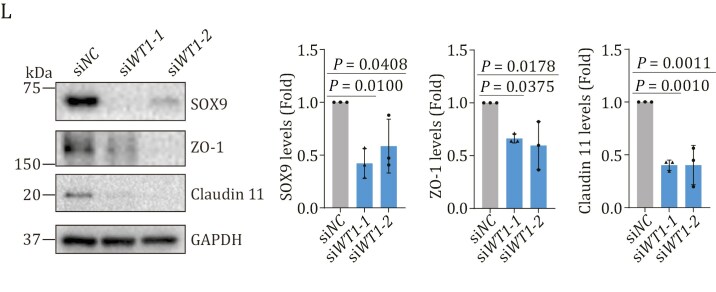


These details have been corrected only in this correction notice to preserve the published version of record

