# Supplementary material for: A single-nucleus transcriptomic atlas of primate testicular aging reveals exhaustion of the spermatogonial stem cell reservoir and loss of Sertoli cell homeostasis
Source: Protein Cell. 2022 Nov 18;14(12):888–907. doi: 10.1093/procel/pwac057 (PMC10691849; doi:10.1093/procel/pwac057)
Supplement: pwac057_suppl_Supplementary_Materials [file pwac057_suppl_supplementary_materials.pdf]

Supplemental Materials

Figure S1 Age-associated phenotypes in the young and aged cynomolgus monkey testes.

(A) Immunohistochemistry analysis of thickness of basement membrane (BM, marked by Collagen IV) in testicular tissues from young and aged monkeys. Representative images are shown on the left. Thickness of BM is quantified as fold changes (Aged vs. Young), and shown as means ± SEM on the right. Young, $n = 4$; aged, $n = 4$ monkeys. Scale bars, 20 μm and 5 μm (Zoomed-in image).

(B) Representative images of co-stainings of SPiDER-βGal, collagen IV, and UTF1 in testicular tissues from aged monkeys. Scale bars, 20 μm and 5 μm (Zoomed-in image).

(C) Representative images of co-stainings of SPiDER-βGal, collagen IV, and WT1 in testicular tissues from aged monkeys. Scale bars, 20 μm and 5 μm (Zoomed-in image).

Figure S2 Single-nucleus transcriptome profiling of young and old cynomolgus monkey testes.

(A) Plots showing the mean reads per nucleus, nucleus number, gene number and unique molecular identifier (UMI) number per nucleus across each testicular sample of young and aged monkeys.

(B) UMAP plots showing the distribution of cells across each sample of monkey testes.

(C) Dot plot showing the expression levels of representative marker genes for each cell type in young and aged monkey testes.

(D) Box plots showing the cell proportion of each sample across different cell types in young and aged monkey testes. The *P*-values of the aged group compared to the young group were calculated using one-sided Wilcoxon Rank Sum test.

Figure S3 Single-nucleus transcriptomic signatures of NHP testicular aging.

(A) Heatmap showing the expression levels of cell type-specific marker genes associated with Aging Atlas database across different cell types in monkey testes.

(B) Dot plot showing genes differentially expressed in at least six cell types.

Figure S4 Dynamic transcriptome programs along the spermatogenesis trajectory in NHP testes.

(A) Pseudotime analysis showing the expression levels of representative genes in gene clusters along the trajectory of spermatogenesis process in monkey testes.

(B) Left, box plot showing the gene set scores for perinuclear theca-related genes in indicated cell types. Right, heatmap showing the expression levels of DEGs associated with perinuclear theca.

(C) Heatmaps showing the expression levels of DEGs associated with indicated gene sets.

(D) Core TFs predicted by SCENIC using pseudotime gene clusters of spermatogonia. Left, network plot showing the core TFs, inter nodes represent core TFs and node size positively correlates with the number of target genes regulated by specific TFs. Each outer node represents one target gene. Right, curve charts showing the gene expression levels of representative core TFs along the pseudotime trajectory of spermatogonia.

(E) Core TFs predicted by SCENIC using aging-associated DEGs of spermatogonia. Left, network plot showing the core TFs. Node size positively correlates with the number of target genes regulated by specific TFs. Right, curve charts showing the gene expression levels of representative core TFs along the pseudotime trajectory of spermatogonia.

Figure S5 Age-dependent transcriptomic alterations in NHP Sertoli cells.

(A) Bar plots showing the enriched GO terms for upregulated and downregulated aging-related DEGs of Sertoli cells.

(B) Heatmaps showing the cell-cell interaction numbers across cell types in young and aged groups of monkey testes.

(C) The top 50 marker genes of each state of Sertoli cells. Marker genes are ranked by LogFC and the rank 1-10 are annotated by gene names.

(D) Violin plots and heatmaps showing the gene set scores and expression levels of aging-related DEGs associated with the indicated gene sets between different states of Sertoli cells in monkey testes, respectively.

Figure S6 Downregulation of a panel of transcription factors in aged Sertoli cells.

(A) Network plot showing the upregulated core TFs predicted by SCENIC using aging-related DEGs of Sertoli cells. Inter nodes represent upregulated core TFs and node size positively correlates with the number of target genes regulated by specific TF. Each outer node represents one target gene.

(B) Western blot analysis of GATA-4 protein expression in testicular tissues from young and aged monkeys. GATA-4 protein levels are quantified as fold changes (Aged vs. Young), and shown as means ± SEM on the right. Young, $n = 4$; aged, $n = 4$ monkeys.

(C) Violin plot showing the *WT1* mRNA expression level between different states of Sertoli cells in monkey testes.

(D) SA-β-Gal staining of human Sertoli cells upon treatment of vehicle or H_2O_2 (100 μM). Representative images are shown on the left. SA-β-Gal-positive cells are quantified as fold changes (H_2O_2 vs. vehicle), and shown as means ± SEM on the right. $n = 3$, three independent replicates.

(E) Western blot analysis of WT1 protein expression in Sertoli cells upon treatment of vehicle or H_2O_2 (100 μM). WT1 protein levels are quantified as fold changes (H_2O_2 vs. vehicle), and shown as means ± SEM on the right. $n = 3$, three independent replicates.

(F) Western blot analysis of WT1 protein expression in Sertoli cells after CRISPR/Cas9-mediated knockdown of *WT1*. Relative protein levels are quantified as fold changes (sg*WT1* vs. sgNC), and shown as means ± SEM on the right. $n = 3$, three independent replicates.

(G) SA-β-Gal staining of Sertoli cells after CRISPR-Cas9-mediated knockdown of *WT1*. Representative images are shown on the left. SA-β-Gal-positive cells are quantified as fold changes (sg*WT1* vs. sgNC), and shown as means ± SEM on the right. $n = 3$, three independent replicates.

(H) Western blot analysis of SOX9 protein expression in testicular tissues from young and aged monkeys. SOX9 protein levels are quantified as fold changes (Aged vs. Young), and shown as means ± SEM on the right. Young, $n = 4$; aged, $n = 4$ monkeys.

(I) Western blot analysis of WT1, SOX9 and ZO-1 protein expression in testicular

tissues from young and aged mice. Indicated protein levels are quantified as fold changes (Aged vs. Young), and shown as means ± SEM on the right. Young, $n = 4$; aged, $n = 4$ mice.

Supplementary Tables

Table S1. Marker genes and cell numbers of each cell type in monkey testes.

Table S2. Aging-related differentially expressed genes of each cell type in monkey testes.

Table S3. Core transcriptional regulators for aging-related differentially expressed genes or differentially expressed genes along the pseudotime of indicated cell types in monkey testes.

Table S4. Gene sets used in this study.

Table S5. Top differentially expressed genes (DEGs) along pseudotime trajectories for germ cells, spermatogonia and Sertoli cells.

Table S6. Cell-cell interaction pairs lost and gained with age between Sertoli cells and other cell types in monkey testes.

Figure S1

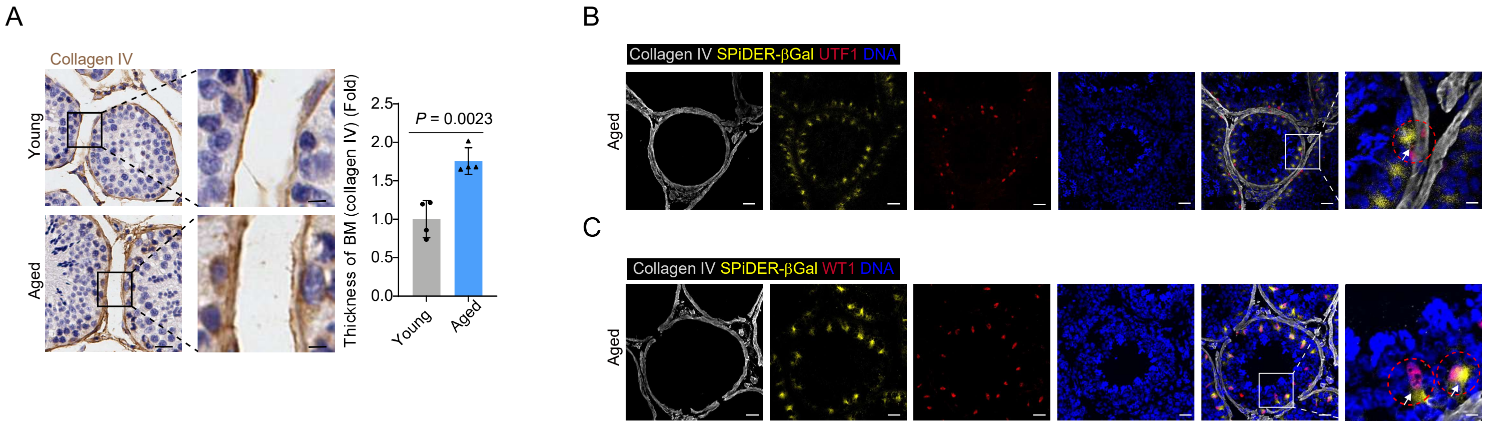
A
Collagen IV
Young
Aged
P = 0.0023
Thickness of BM (collagen IV) (Fold)
2.5
2.0
1.5
1.0
0.5
0.0
Young
Aged
B
Collagen IV SPiDER-βGal UTF1 DNA
Aged
C
Collagen IV SPiDER-βGal WT1 DNA
Aged

Figure S2

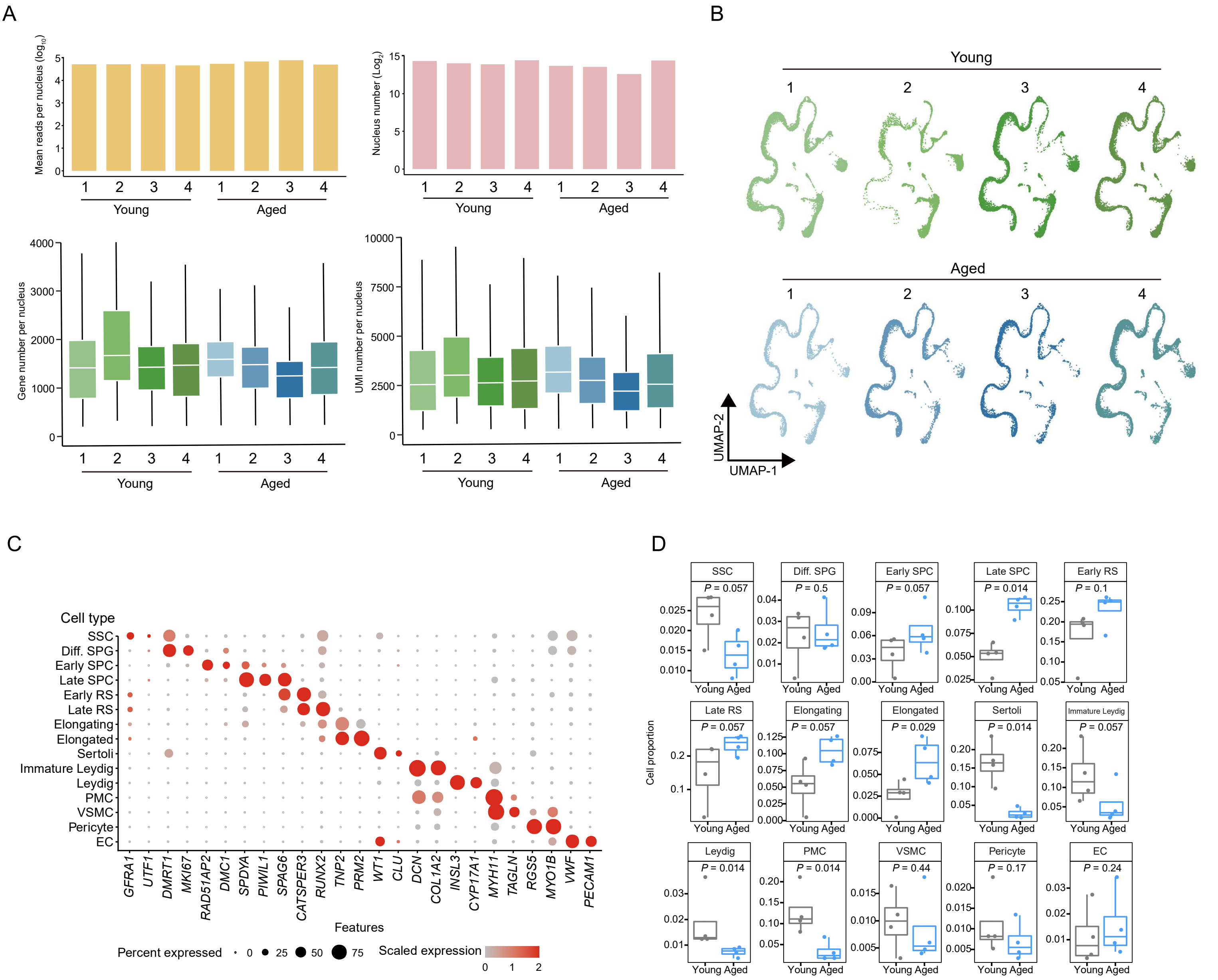

A
Mean reads per nucleus (log10)
Nucleus number (Log2)
Gene number per nucleus
UMI number per nucleus
1 2 3 4 1 2 3 4
Young
Aged
B
Young
Aged
UMAP-2
UMAP-1
C
Cell type
SSC
Diff. SPG
Early SPC
Late SPC
Early RS
Late RS
Elongating
Elongated
Sertoli
Immature Leydig
Leydig
PMC
VSMC
Pericyte
EC
GFRA1 UTF1 DMRT1 MKI67 RAD51AP2 DMC1 SPDYA PIWIL1 SPAG6 CATSPER3 RUNX2 TNP2 PRM2 WT1 CLU DCN COL1A2 INSL3 CYP17A1 MYH11 TAGLN RGS5 MYO1B VWF PECAM1
Features
Percent expressed 0 25 50 75
Scaled expression 0 1 2
D
Cell proportion
SSC P = 0.057
Diff. SPG P = 0.5
Early SPC P = 0.057
Late SPC P = 0.014
Early RS P = 0.1
Late RS P = 0.057
Elongating P = 0.057
Elongated P = 0.029
Sertoli P = 0.014
Immature Leydig P = 0.057
Leydig P = 0.014
PMC P = 0.014
VSMC P = 0.44
Pericyte P = 0.17
EC P = 0.24
Young Aged

Figure S3

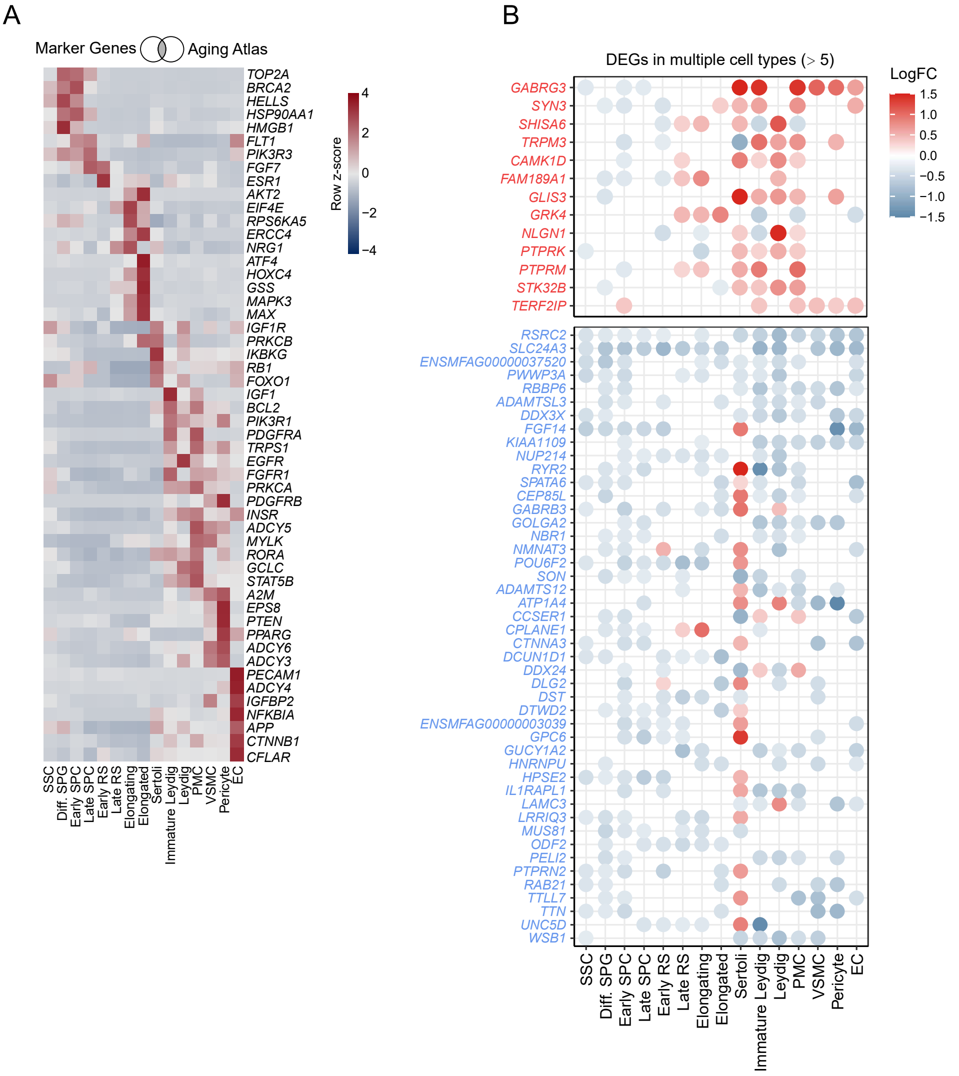

A
Marker Genes
Aging Atlas
Row z-score
B
DEGs in multiple cell types (> 5)
LogFC
SSC
Diff. SPG
Early SPC
Late SPC
Early RS
Late RS
Elongating
Elongated
Sertoli
Immature Leydig
Leydig
PMC
VSMC
Pericyte
EC

Figure S4

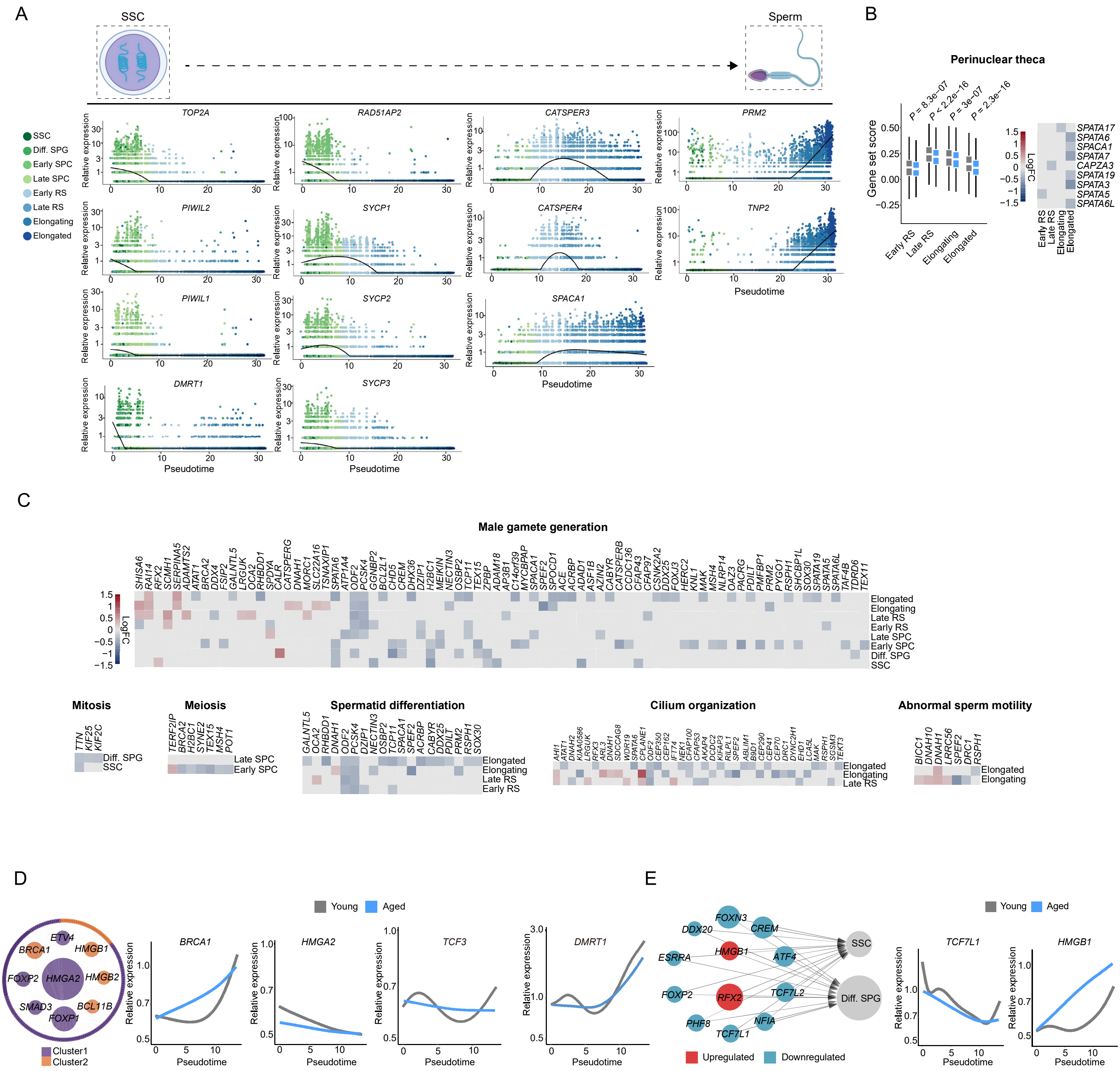
A
SSC
Sperm
SSC
Diff. SPG
Early SPC
Late SPC
Early RS
Late RS
Elongating
Elongated
TOP2A
RAD51AP2
CATSPER3
PRM2
PIWIL2
SYCP1
CATSPER4
TNP2
PIWIL1
SYCP2
SPACA1
DMRT1
SYCP3
Relative expression
Pseudotime
B
Perinuclear theca
Gene set score
P = 8.3e−07
P < 2.2e−16
P = 3e−07
P = 2.3e−16
Early RS
Late RS
Elongating
Elongated
LogFC
SPATA17
SPATA6
SPACA1
SPATA7
CAPZA3
SPATA19
SPATA3
SPATA5
SPATA6L
C
Male gamete generation
Mitosis
Meiosis
Spermatid differentiation
Cilium organization
Abnormal sperm motility
D
Young
Aged
Cluster1
Cluster2
ETV4
BRCA1
HMGB1
FOXP2
HMGA2
HMGB2
SMAD3
BCL11B
FOXP1
BRCA1
HMGA2
TCF3
DMRT1
E
FOXN3
DDX20
CREM
SSC
ESRRA
HMGB1
ATF4
FOXP2
RFX2
TCF7L2
Diff. SPG
PHF8
NFIA
TCF7L1
Upregulated
Downregulated
TCF7L1
HMGB1

Figure S5

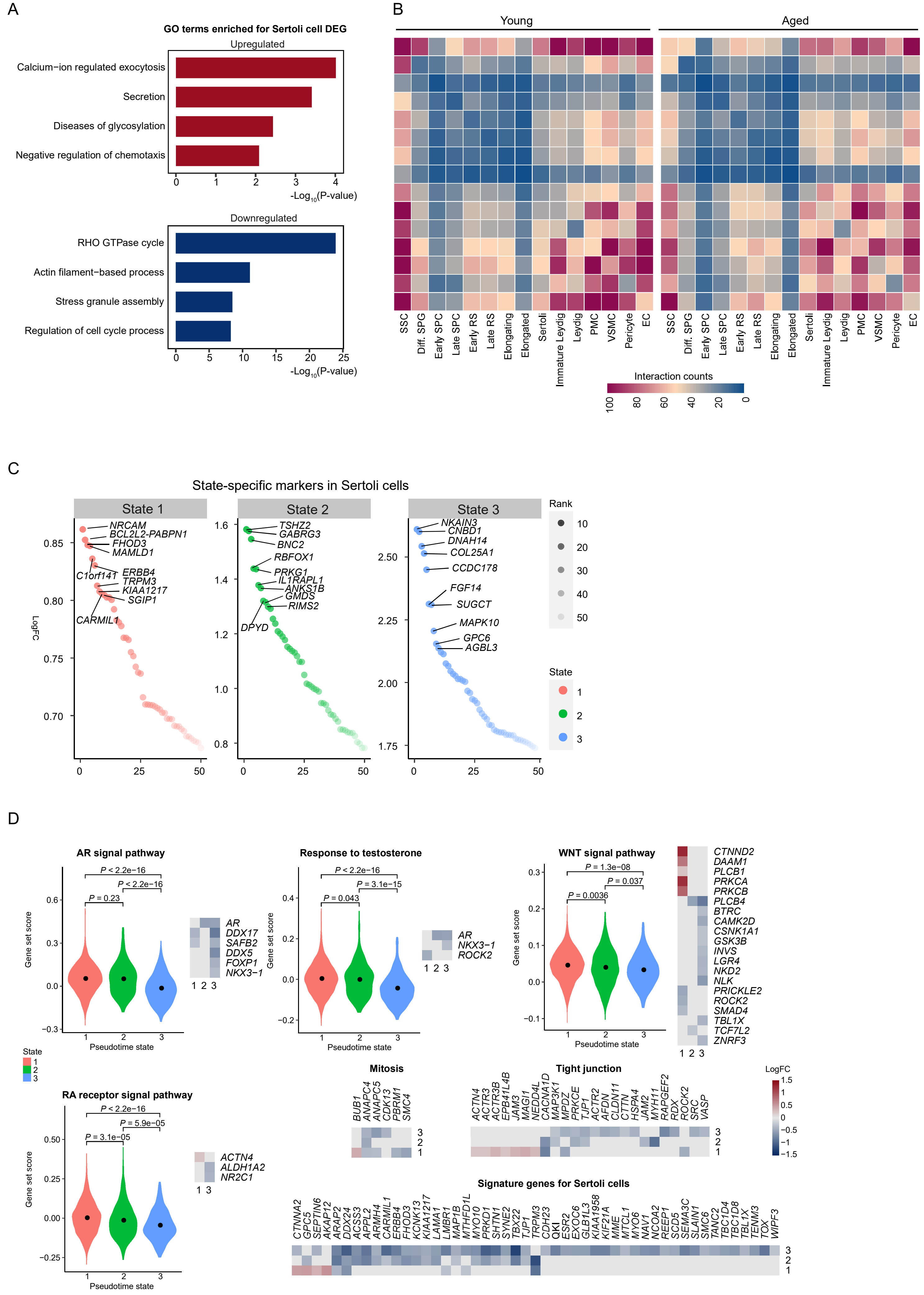
A
GO terms enriched for Sertoli cell DEG
Upregulated
Calcium−ion regulated exocytosis
Secretion
Diseases of glycosylation
Negative regulation of chemotaxis
-Log10(P-value)
Downregulated
RHO GTPase cycle
Actin filament−based process
Stress granule assembly
Regulation of cell cycle process
-Log10(P-value)
B
Young
Aged
SSC
Diff. SPG
Early SPC
Late SPC
Early RS
Late RS
Elongating
Elongated
Sertoli
Immature Leydig
Leydig
PMC
VSMC
Pericyte
EC
Interaction counts
C
State-specific markers in Sertoli cells
State 1
State 2
State 3
LogFC
NRCAM
BCL2L2-PABPN1
FHOD3
MAMLD1
C1orf141
ERBB4
TRPM3
KIAA1217
SGIP1
CARMIL1
TSHZ2
GABRG3
BNC2
RBFOX1
PRKG1
IL1RAPL1
ANKS1B
GMDS
RIMS2
DPYD
NKAIN3
CNBD1
DNAH14
COL25A1
CCDC178
FGF14
SUGCT
MAPK10
GPC6
AGBL3
Rank
State
D
AR signal pathway
Response to testosterone
WNT signal pathway
RA receptor signal pathway
Gene set score
Pseudotime state
P < 2.2e−16
P = 0.23
P = 0.043
P = 3.1e−15
P = 1.3e−08
P = 0.037
P = 0.0036
P = 3.1e−05
P = 5.9e−05
AR
DDX17
SAFB2
DDX5
FOXP1
NKX3−1
ROCK2
CTNND2
DAAM1
PLCB1
PRKCA
PRKCB
PLCB4
BTRC
CAMK2D
CSNK1A1
GSK3B
INVS
LGR4
NKD2
NLK
PRICKLE2
SMAD4
TBL1X
TCF7L2
ZNRF3
ACTN4
ALDH1A2
NR2C1
Mitosis
BUB1
ANAPC4
ANAPC5
CDK13
PBRM1
SMC4
Tight junction
ACTN4
ACTR3
ACTR3B
EPB41L4B
JAM3
MAGI1
NEDD4L
CACNA1D
MAP3K1
MPDZ
PRKCE
TJP1
ACTR2
AFDN
CLDN11
CTTN
HSPA4
JAM2
MYH11
RAPGEF2
RDX
ROCK2
SRC
VASP
LogFC
Signature genes for Sertoli cells
CTNNA2
GPC5
SEPTIN6
AKAP12
ARAP2
DDX24
ACSS3
APPL2
ARMH4
CARMIL1
ERBB4
FHOD3
KCNK13
KIAA1217
LAMA1
LMBR1
MAP1B
MTHFD1L
MYO10
PRKD1
SHTN1
SYNE2
TBX22
TJP1
TRPM3
CDH23
QKI
ESR2
EXOC6
GLB1L3
KIAA1958
KIF21A
MME
MTCL1
MYO6
NAV1
NCOA2
REEP1
SCD5
SEMA3C
SLAIN1
SMC6
TANC2
TBC1D4
TBC1D8
TBL1X
TENM3
TOX
WIPF3

Figure S6

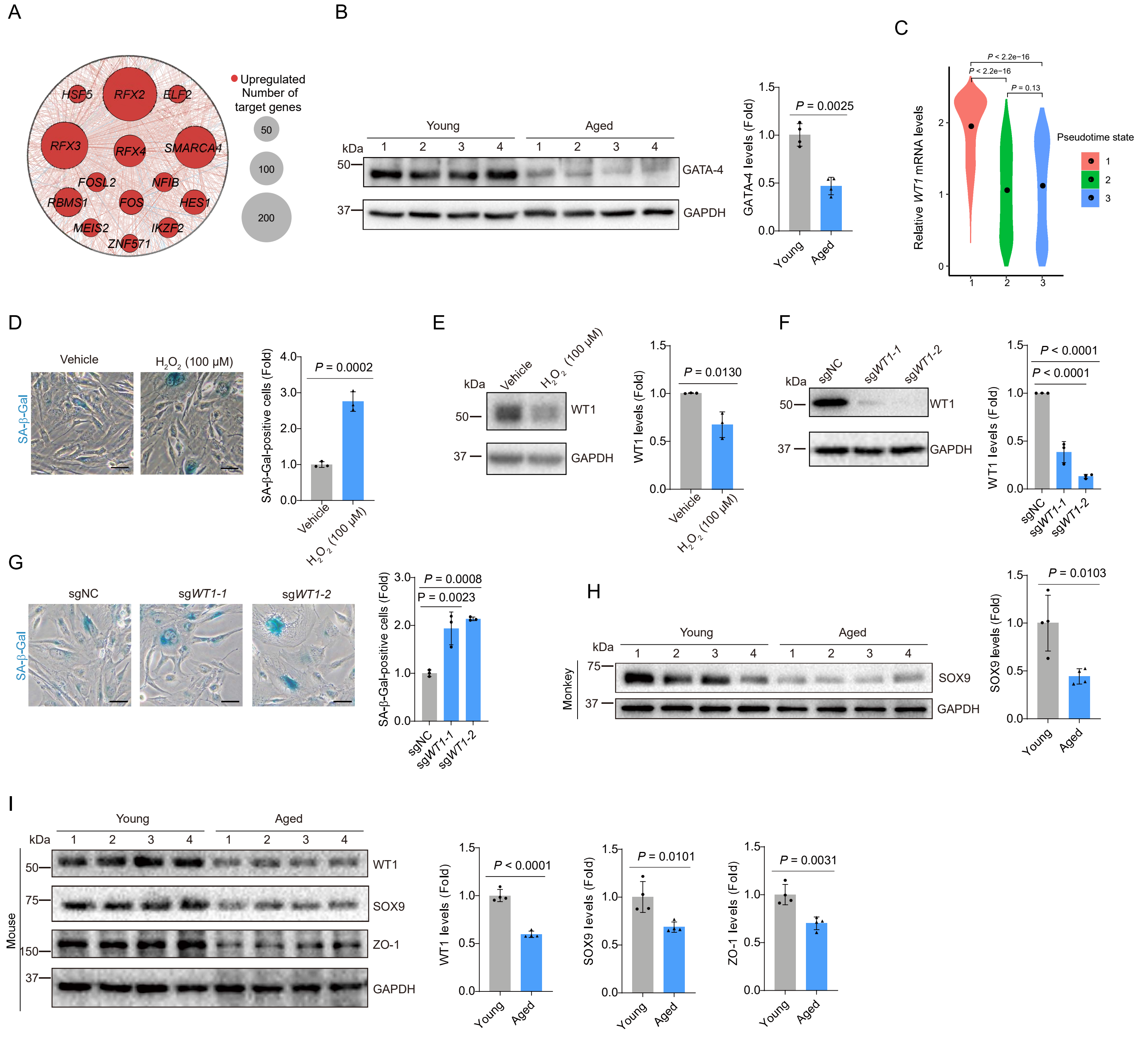
A
B
C
D
E
F
G
H
I
Upregulated
Number of target genes
50
100
200
HSF5
RFX2
ELF2
RFX3
RFX4
SMARCA4
FOSL2
NFIB
RBMS1
FOS
HES1
MEIS2
IKZF2
ZNF571
Young
Aged
kDa
GATA-4
GAPDH
GATA-4 levels (Fold)
P = 0.0025
Relative WT1 mRNA levels
P < 2.2e−16
P = 0.13
Pseudotime state
Vehicle
H2O2 (100 μM)
SA-β-Gal
SA-β-Gal-positive cells (Fold)
P = 0.0002
WT1
WT1 levels (Fold)
P = 0.0130
sgNC
sgWT1-1
sgWT1-2
P < 0.0001
P = 0.0008
P = 0.0023
Monkey
SOX9
SOX9 levels (Fold)
P = 0.0103
Mouse
ZO-1
ZO-1 levels (Fold)
P < 0.0001
P = 0.0101
P = 0.0031
